# Target Analysis Resolves the Ground and Excited State Properties from Femtosecond Stimulated Raman Spectra

**DOI:** 10.1021/acs.jpclett.4c01555

**Published:** 2024-09-06

**Authors:** Ivo H.M. van Stokkum, Joris J. Snellenburg, Petra Chrupková, Jakub Dostal, Sebastian Weigand, Jörn Weißenborn, John T.M. Kennis, Miroslav Kloz

**Affiliations:** † Department of Physics and Astronomy and LaserLaB, Faculty of Science, Vrije Universiteit Amsterdam, De Boelelaan 1081, 1081 HV Amsterdam, The Netherlands; ‡ The Extreme Light Infrastructure ERIC, ELI Beamlines Facility, Za Radnicí 835, 252 41 Dolní Břežany, Czech Republic

## Abstract

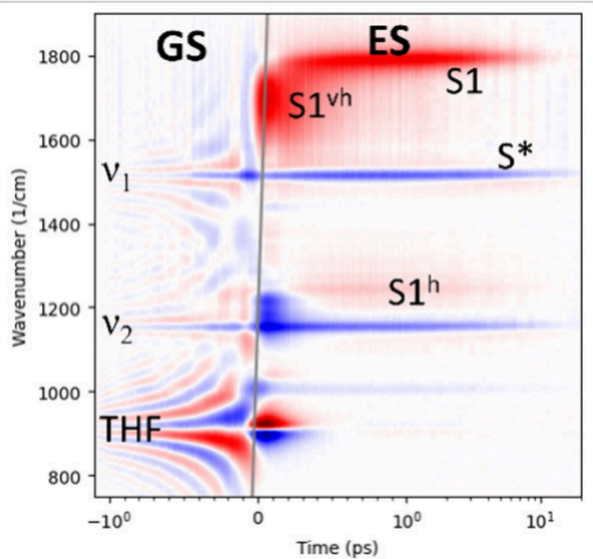

Target analysis is employed to resolve the ground and excited state properties from simultaneously measured Femtosecond Stimulated Raman Spectra (FSRS) and Transient Absorption Spectra (TAS). FSRS is a three-pulse technique, involving picosecond Raman pump pulses and femtosecond visible pump and probe pulses. The TAS are needed to precisely estimate the properties of the Instrument Response Function. The prezero “coherent artifact” present during the overlap of the three pulses is described by a damped oscillation with a frequency (ω – *ω*_*n*_) where *ω*_*n*_ is a ground state resonance Raman frequency. Simultaneous target analysis of the FSRS and TAS allows the complete excited state dynamics to be resolved with a time resolution better than 100 fs. The model system studied is the carotenoid lycopene in tetrahydrofuran. The lycopene dynamics show a spectral evolution with seven states, including a biphasic cooling process during the S2–S1 internal conversion, multiple S1 lifetimes, and an S* state decaying with a lifetime of 7 ps.

Time-resolved experiments, employing both Femtosecond Stimulated Raman Spectroscopy (FSRS) and Transient Absorption (TA), contain a wealth of information.^1−7^ To extract this information, in particular the ground and excited state properties of the solvated chromophore under study, a target model-based parametric description of the data is mandatory.^8^ FSRS is a three-pulse technique, it involves the use of picosecond Raman pump pulses (E_R_ in Figure 1A) overlapped with femtosecond white-light continuum probe pulses and an excitatory visible pump pulse (E_P_ and E_A_ in Figure 1A). Particularly challenging is the treatment of the “coherent artifact” (CA) present during the overlap regions of the three pulses.^9^ Below we will demonstrate that this CA can be described by a damped oscillation with a frequency (ω – *ω*_*n*_) where *ω*_*n*_ is a resonance frequency of the ground state (GS) of the chromophore or of the solvent.^10^ We chose as a model system for the development of our FSRS target analysis methodology the unsubstituted “simple” carotenoid lycopene^11,12^ in tetrahydrofuran (THF) (structures in Figure S1) although even this “simple” carotenoid can exhibit complex behavior when aggregated.^13,14^ Thus, a detailed characterization of the photophysics of the lycopene monomer is a prerequisite for understanding the more complex behavior in aggregates. Recently, neurosporene, spheroidene and lycopene (with 9, 10, and 11 conjugated double bonds, respectively) have been measured in cyclohexane.^15^ An S* state was only resolved in lycopene, which provides another incentive for a more detailed investigation of the relaxation pathways in lycopene.

**Figure 1:**
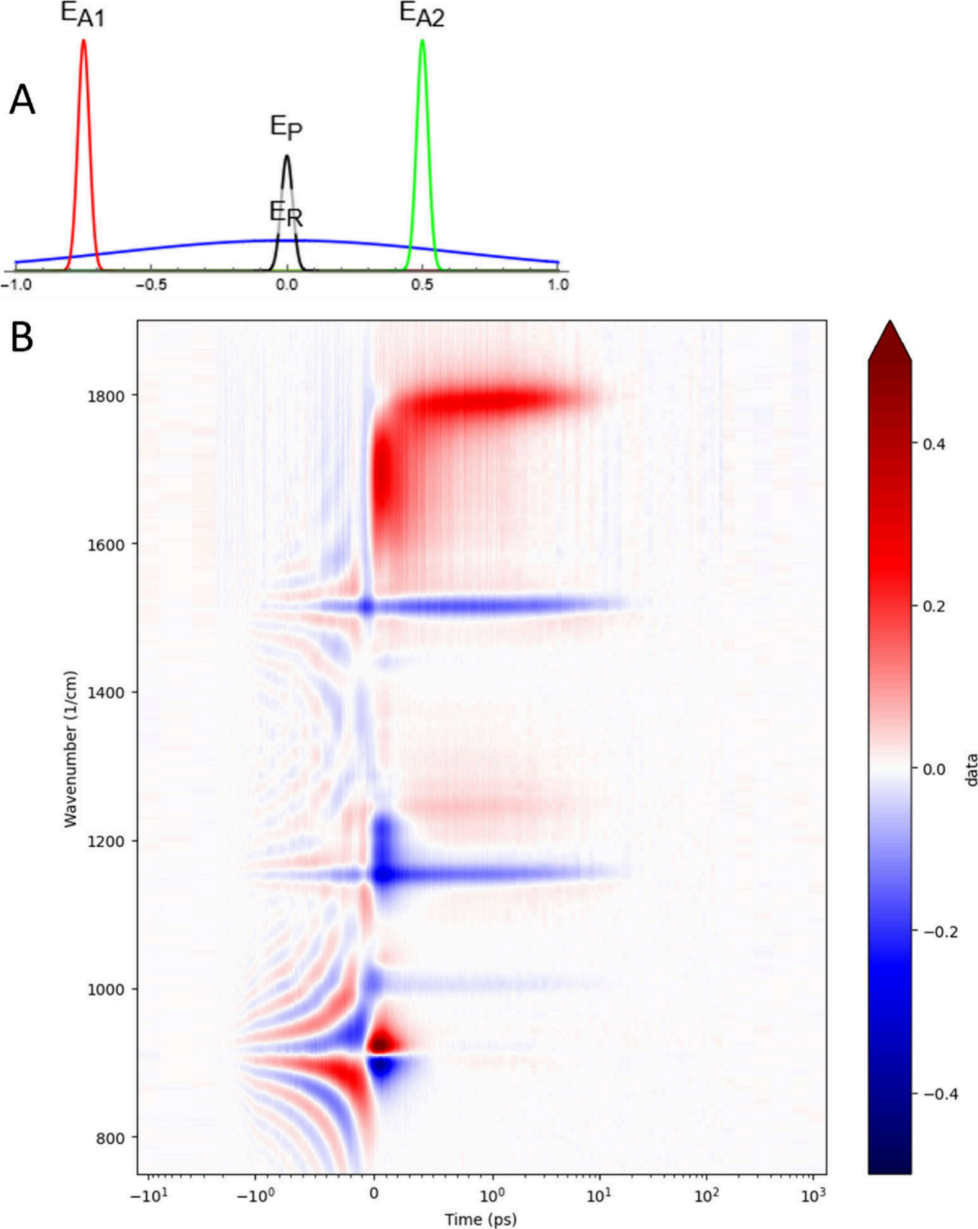
(A) Approximate shapes and positions of the picosecond Raman pump pulse (E_R_, blue), the white-light continuum probe pulse (E_P_, black), and the excitatory visible pump pulses (E_A1_ and E_A2_) at pump–probe delays of 0.75 (red) and −0.5 ps (green). (B) Time-resolved Stokes FSRS difference spectrum of lycopene in THF (in mOD) after excitation at 480 nm, using a ≈790 nm Raman pump. Note that the time axis is linear from −1 to 1 ps, and logarithmic elsewhere.

Resonance Raman spectra of carotenoids contain four characteristic bands, labeled ν_1_ to ν_4_,^16−18^ which provide detailed vibrational information about the ground state. The ν_1_ region, representing the C=C vibration between 1510 and 1530 cm^–1^, acts as a signature for the C=C stretch mode. Notably, it experiences a substantial upshift in the excited state (ES). The ν_2_ band at 1160 cm^–1^ encompasses contributions from stretching vibrations of C–C single bonds coupled with C–H in-plane bending modes, serving as a distinctive feature for carotenoid configurations, especially isomerization states. The ν_3_ band at 1000 cm^–1^ arises from in-plane rocking vibrations of methyl groups attached to the conjugated chain, coupled with in-plane bending modes of adjacent C–H’s, functioning as a signature for the conjugated end-cycle configuration. The ν_4_ band at 960 cm^–1^ results from C–H out-of-plane wagging motions coupled with C=C torsional modes. When the carotenoid conjugated system is planar, which is the case with lycopene, these out-of-plane modes are uncoupled from the electronic transition, rendering these bands non-Raman active. With the help of the target analysis of FSRS we can monitor the dynamics of the ν_1_ to ν_3_ bands in the ES.

The Stokes FSRS difference spectrum of lycopene in THF in Figure 1B shows that before time zero strong resonance-like signals are present which resemble a perturbed free induction decay (PFID) of the probe pulse electric field in the presence of the Raman pump pulse, which is halted by the arrival of the excitatory visible pump pulse.^10^ After excitation the time-resolved Stokes FSRS difference spectrum shows a complicated spectral evolution. Visual inspection of Figure 1B shows striking zero crossings around wavenumbers of ≈900, ≈1150, and ≈1500 cm^–1^. The time resolution of the experiment is determined by the Instrument Response Function (IRF), which is the convolution of the visible pump and probe pulse, and typically is ≈180 fs fwhm. The solvent THF has a strong Raman band at ≈913 cm^–1^.^19^ Thus, the prezero signals around 913 cm^–1^ can be attributed to the solvent, whereas the prezero signals around ≈1500, and ≈1150 cm^–1^ can be attributed to the ν_1_ and ν_2_ bands of lycopene GS, respectively. Note that during the overlap of the pump and the probe pulses the solvent shows an intense dispersive signal around the Raman line maximum,^9^ which is also visible in Figure 1B around 0 ps and 913 cm^–1^. In the absence of the Raman pump pulse, this is an “ordinary” pump–probe transient absorption experiment, providing us with TAS data. In addition to the huge FSRS signals around 0 ps (Figure 1B) the TAS provide independent information on the (wavenumber-dependent) location of the maximum of the IRF and its width, and of the spectral evolution of the ES. Thus, we commence with the description of the global and target analysis of TAS in the visible of lycopene in THF to establish a putative kinetic scheme, which can then be employed for the target analysis of the FSRS data. Note that all parameters are estimated from a simultaneous analysis of all data (as detailed in the Methods).

First, a sequential kinetic scheme without losses (Figure 2A) has been employed to describe the TAS data. The IRF width of the dedicated TA setup was ≈97 fs fwhm. Six components are needed for a satisfactory fit (Figure S2, Figure S3, Figure S4). The concentrations are depicted in Figure 2B. The estimated lifetimes are 71, 175 fs, 0.8, 3.3, and 5.9 ps, and long-lived. The visible TA EADS in Figure 2C summarize the spectral evolution. The negative of the ground state absorption spectrum is shown in orange in (Figure 2C,F), demonstrating the extrema of the bleach around 450, 480, and 510 nm. Note that in the EADS (Figure 2C) which are estimated using a sequential scheme without losses (Figure 2A) the bleach around 500 nm shows three main decay phases, 0.8, 3.3, and 5.9 ps (from blue to green, from green to magenta, and from magenta to cyan, respectively). The 3.3 ps EADS (green) peaks at 565 nm, which is interpreted as the excited state absorption (ESA) of the relaxed S1 state. However, the 5.9 ps EADS (magenta) also peaks at 565 nm, indicating a more slowly decaying subpopulation of S1. Therefore, in the target analysis, we employ a kinetic scheme with several loss channels (Figure 2D): during the 0.8 ps phase 25% of blue decays to the ground state (GS), during the ≈3 ps phase 14% of relaxed S1 decays to a subpopulation of S1 with identical SADS decaying with a lifetime of 5 ps, and the majority (64%) decays to a state called S* that peaks at ≈545 nm and decays with a lifetime of 6.7 ps. The triplet state (T) is formed from both S1 populations with a small rate of 5 ns^–1^. With this kinetic scheme, the bleach amplitudes around 450, 480, and 510 nm of the SADS (red, blue, green, magenta and cyan in Figure 2F) are now more comparable. In addition, also the ESA bands around 565 nm of the blue and green SADS are now more comparable. The fit quality of this target analysis is comparable to that of the global analysis since the amount of different SADS is the same as the amount of EADS (both 6).

**Figure 2:**
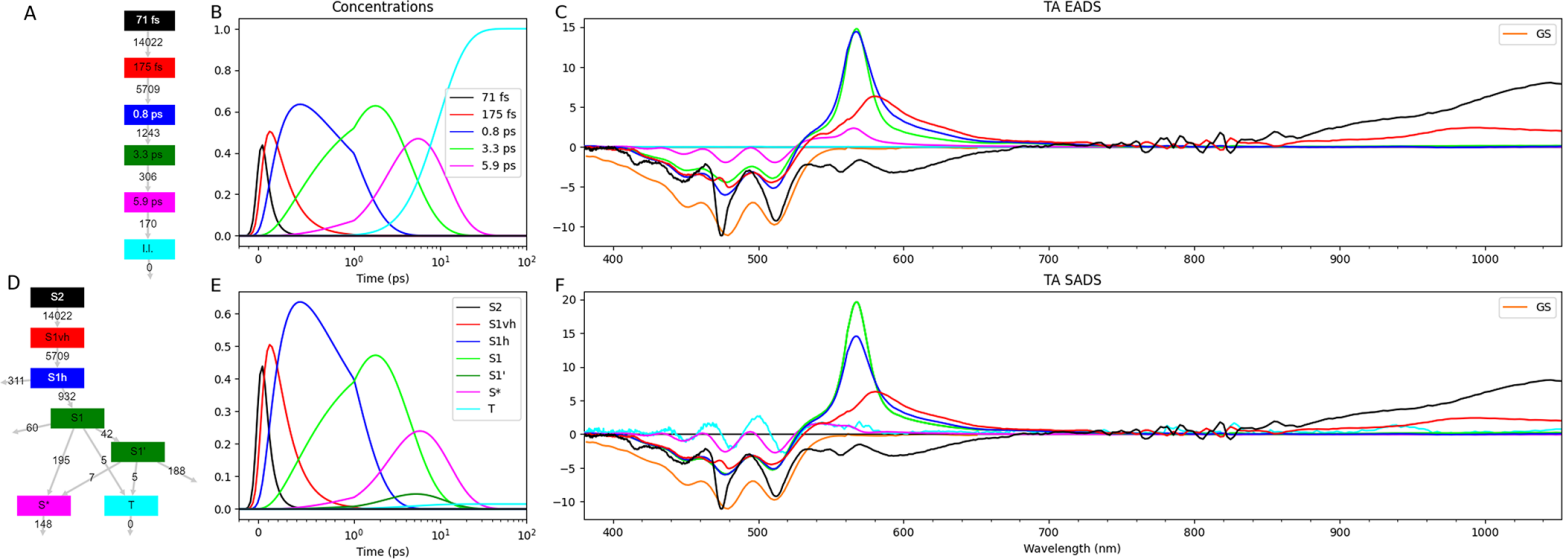
Global and target analysis of TAS of lycopene in THF with OD_480 nm_ equal to 0.1. Concentrations (B,E) of a sequential scheme without losses (A) and of a target kinetic scheme (D) with rate constants in ns^–1^. Legends in (B,E) indicate lifetimes and species, l.l. is long-lived; S1vh is very hot S1, S1h is hot S1, S1′ is a more slowly decaying subpopulation of S1 with identical SADS, T is triplet. Estimated EADS (C) and SADS (F, both in mOD) using the kinetic schemes from A and D. The negative of the scaled ground state absorption spectrum is shown in orange in (C,F).

Three different samples of lycopene in THF have been measured with OD_480 nm_ equal to 0.1, 0.3, and 1.0, and the results were consistent (Figure S3). There were no signs of aggregation at OD_480 nm_ equal to 1.0, which was the OD used with the FSRS experiments to achieve a sufficient Signal-to-Noise Ratio (SNR). All TAS data could be well fitted with the same kinetic scheme and lifetimes as in Figure 2 (Figure S3). The region around 800 nm is problematic in Figure 2C,F because the white light is generated from an 800 nm beam. The SADS estimated from the visible TA data of the FSRS experiments (Figure S6D) are generally consistent with the SADS in Figure 2F, but they are smooth around 800 nm.

The first SADS (black in Figure 2F, Figure S6D) can be interpreted as the S2 state, which with a lifetime of 71 fs decays to a very hot S1 state (red in Figure 2F, Figure S6D). Whereas the S2 SADS has bleach below 520 nm, stimulated emission (SE) from ≈530 to ≈700 nm, and a large broad ESA above 700 nm, the very hot S1 SADS has ESA around 970 nm next to the well-known ESA from ≈540–750 nm. Next, the hot S1 state (blue in Figure 2F, Figure S6D) rises with a lifetime of 174 fs and decays with a lifetime of 1.63 ps. Its ESA extends from ≈520–750 nm with a narrow peak around 565 nm. The relaxed S1 state (green) rises with a lifetime of 0.8 and mainly decays with a lifetime of 3.3 ps, but a subpopulation (14%) decays with a lifetime of 5 ps. The S* state (magenta) rises with a lifetime of 3.3 and decays with a lifetime of 6.7 ps. Its SADS has positive difference absorption at ≈545 nm and structured bleach bands (Figure 2F, Figure S6D). The long-lived component (cyan) could possibly be attributed to a very small amount of triplet state.

A concise overview of the global analysis of all data of the FSRS plus TA experiments (with the concentrations and all EADS) is shown in Figure S7. With a Raman excitation wavelength of ≈790 nm the Stokes range 600–1900 cm^–1^ corresponds to ≈829–930 nm, and the Anti-Stokes range 600–1900 cm^–1^ corresponds to ≈687–754 nm. The TAS in the FSRS ranges are well fitted (Figure S8, Figure S9, Figure S10, Figure S11). Note the excellent fit around time zero in Figure S9, which has been achieved through the relatively high weight of these TA data. Thus, the properties of the IRF are precisely estimated. The common IRF width parameter estimate was ≈180 fs fwhm. The black and red TA EADS in Figure S7G,H and I,J are largely consistent. Thus, we can conclude that the wavelength and wavenumber dependence of the maximum of the IRF is largely consistent (cf. the black dispersion curves in Figure S4, Figure S10, Figure S11). Note the agreement between the black and red TA EADS in Figure S7G,I. The black Anti-Stokes TA EADS Figure S7H,J is probably compromised by an imprecise dispersion estimate, but the red curves agree well. Next, we apply a target analysis, zoom in and describe the FSRS SADS estimated with the kinetic scheme of Figure 2D.

For all presented Raman data, both Stokes gain and anti-Stokes loss are plotted as positive. Following the TA convention of absorption being positive and stimulated emission or bleach being negative, the graph depicting FSRS in the Stokes region can be considered flipped to visualize Raman gain as positive. Since Stokes Raman gain provides essentially the same information as anti-Stokes Raman loss, and Raman data are usually visualized as positive in the literature, such a choice is considered natural. However, in raw data, Stokes and anti-Stokes signals are of opposite sign. The fit of the FSRS data is excellent, with only small residuals at larger negative delays (Figure 3, Figure S12, Figure S13, Figure S14). Note that the prezero patterns are very well reproduced by our model function employing the damped oscillation cos((ω-*ω*_*n*_)*t*-*φ*_*n*_ (ω))exp(-*γ*_*n*_*t*) with only three resonance frequencies: ≈913 cm^–1^ (attributed to THF, Figure S15), and 1511 and 1154 cm^–1^ that can be attributed to the ν_1_ and ν_2_ bands of the lycopene GS (Figure S16). Note further that at positive delays bleaches (blue) are present of the ν_1_ and ν_2_ bands of the lycopene GS. As a consequence of the above convention the sign of the THF CA around ≈913 cm^–1^ is inverted (Figure 3A,D). The unnormalized and normalized FSRS SADS in the Stokes and Anti-Stokes regions (Figure 4) shed more light on the complicated spectral evolution of the lycopene ES. Clear bleaches due to the population of the lycopene ES are visible around ≈1520 cm^–1^ (ν_1_), ≈1160 cm^–1^ (ν_2_), ≈1010 cm^–1^ (ν_3_). The bleach of ν_4_ which is expected around ≈960 cm^–1^ is not present, which is to be expected for a linear carotenoid.^16^ The excited states show a gradual upshift of the ν_1_ band, which arises from the stretching vibrations of the conjugated C=C double bonds. The S2 SADS (black curves) most probably still contains some CA. Nevertheless, excess absorption around ≈1800 cm^–1^ appears to be present, especially in the Anti-Stokes FSRS SADS (Figure 4D). The very hot S1 SADS (red curves) shows a very broad excess absorption from ≈1520–1770 cm^–1^ and a negative peak around ≈1800 cm^–1^ in the Anti-Stokes FSRS SADS (Figure 4D), which may be attributable to inverse Raman. The peak of the ESA of the hot S1 SADS (blue curves) is shifted to higher energy, ≈1780 cm^–1^. In addition, excess absorption in the Stokes ν_2_ region is visible around ≈1260 cm^–1^. Thus, we find that the two phases of vibrational cooling with lifetimes of 175 fs and 0.8 ps, visible in the red and the blue SADS in Figure 2F and Figure 4C,D, distinctly differ. A narrower ν_1_ peak of the ESA of the S1 SADS (green curves in Figure 4C,D) is centered around ≈1800 cm^–1^. The fifth normalized EADS (magenta curves in Figure S13E and S14E) show a smaller peak at ≈1800 cm^–1^, indicating the more slowly decaying subpopulation of relaxed S1. The target analysis again successfully resolved the fifth SADS (magenta curves), tentatively assigned to S*, which shows a downshift of the ν_1_, ν_2_, and ν_3_ bands. Recall that the fine structure in the TA bleach was also very pronounced (magenta curves in Figure 2F). The small and noisy cyan SADS, tentatively assigned to a very small amount of triplet state, shows a ν_1_ bleach at ≈1520 cm^–1^ and a dispersive feature around the 913 cm^–1^ THF resonance. Note that all SADS show dispersive features around the 913 cm^–1^ THF resonance. This suggests that the excited carotenoid state interacts with the solvent, possibly dissipating excess energy.

**Figure 3:**
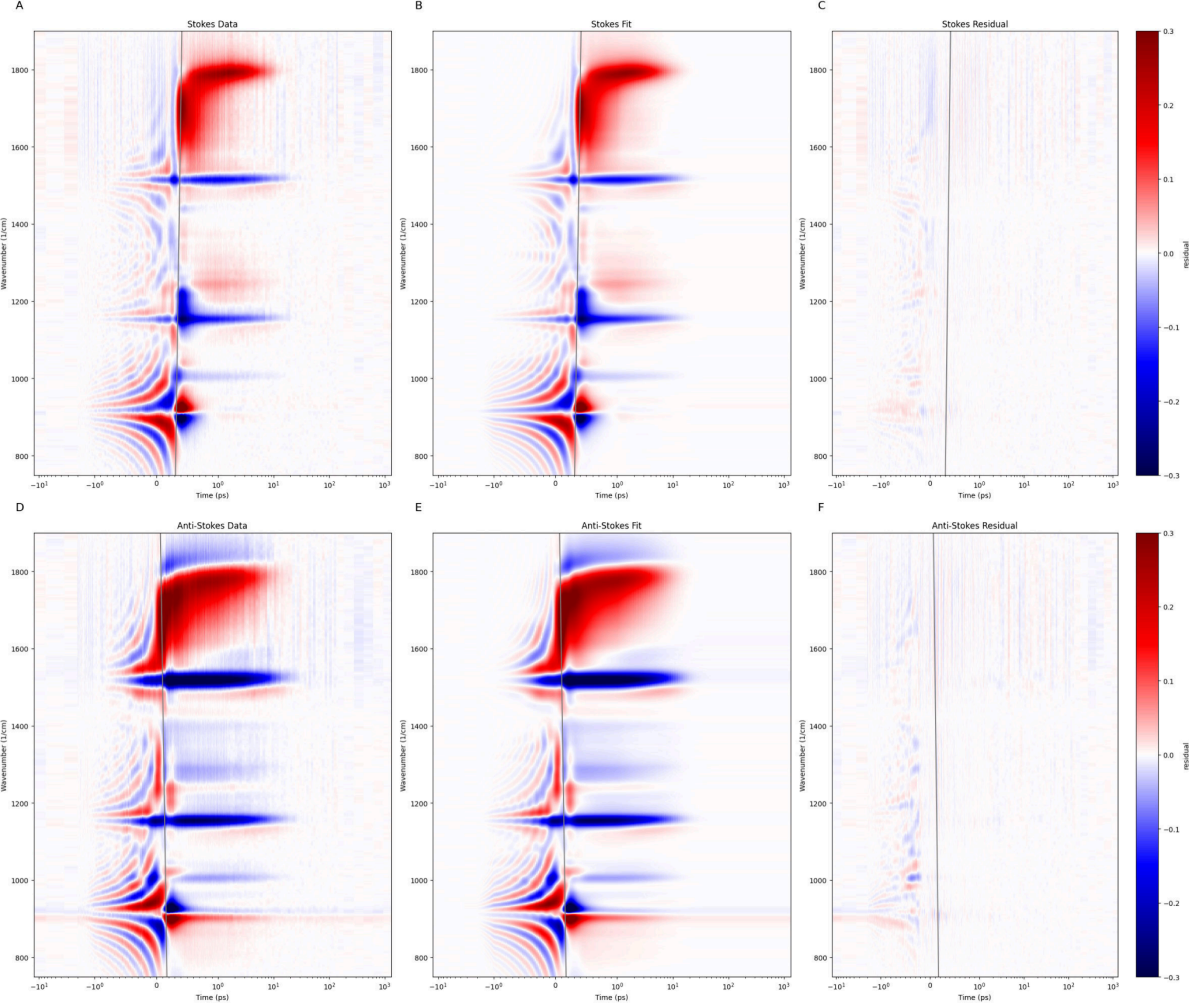
Target analysis of Stokes and Anti-Stokes of FSRS of lycopene in THF (in mOD), note the qualitative and quantitative agreement. From left to right: data, fit and residual. In gray the estimated dispersion curves (the location of the maximum of the IRF). Note that the time axis is linear from −1 to 1 ps and logarithmic elsewhere.

**Figure 4:**
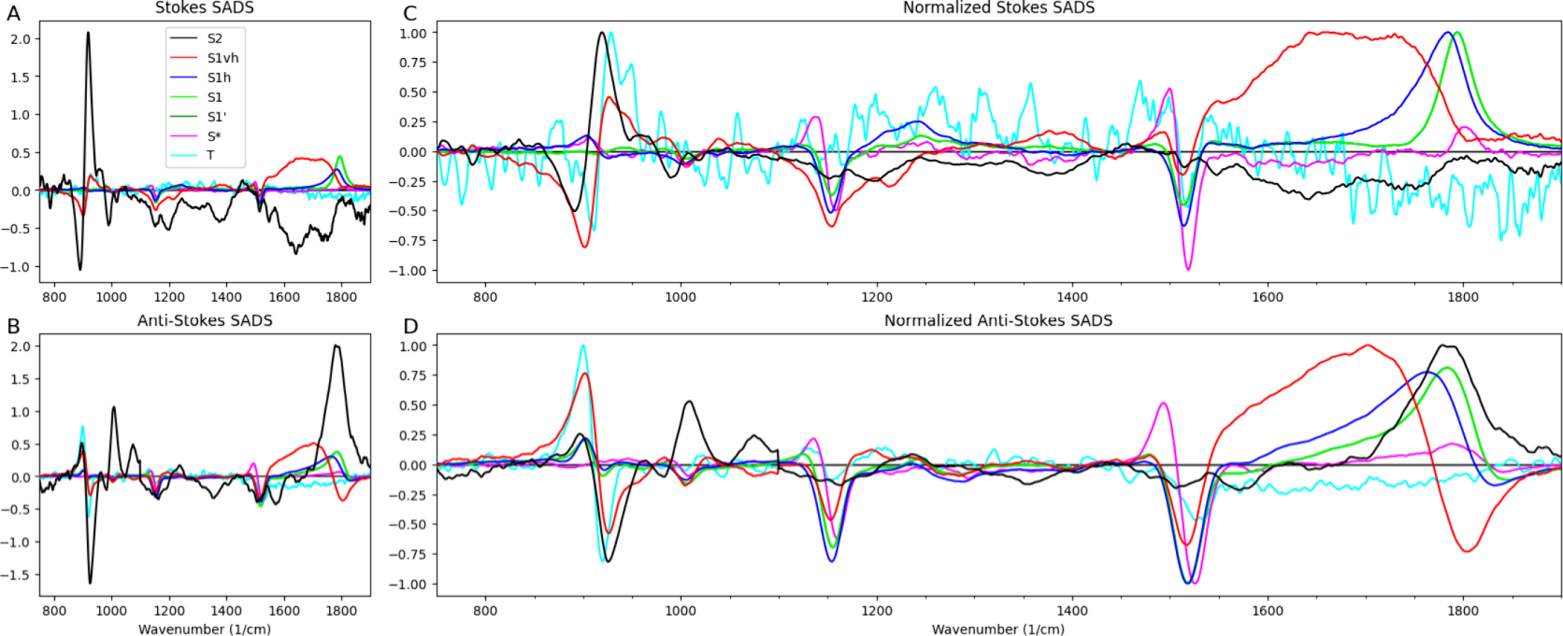
Stokes and Anti-Stokes FSRS SADS (A,B) and normalized SADS (C,D) estimated from the target analysis of lycopene in THF using the kinetic scheme of Figure 2D. Legend in (A) applies to all panels and indicates the species, S1vh is very hot S1, S1h is hot S1, S1′ is a more slowly decaying subpopulation of S1 with identical SADS, T is triplet.

The nature of the S* state has long been a subject of intense debate.^5,20,21^ A model explained S* by vibronic transitions on either S1, S0, or both, depending on the chain length of the investigated carotenoid.^22^ Thus, simultaneous target analysis of the FSRS and TAS of carotenoids of different chain lengths, and with different substituents, is expected to shed more light on this complex question. Recently, in^15^ the downshift of the ν_1_, ν_2_, and ν_3_ bands of the S* state in lycopene (in cyclohexane) is attributed to a downshifted *ground state* C=C mode. The SNR of the present data of lycopene in THF is higher, which allows us to establish the kinetic scheme including the multiexponential features of the relaxations. From our target analysis which includes a longer-lived subpopulation S1′ of the S1 excited state (Figure 2D) this can be confirmed. Nevertheless, in the S* FSRS SADS (magenta in Figure 4C,D) small features of the stretched C=C mode of the excited state around 1800 cm^–1^ remain present. If S* would be a ground state intermediate, then this S* FSRS SADS is not yet “pure” enough, and these features could be attributed to a small amount of the S1 excited state decaying with a lifetime of ≈7 ps, else S* could still be an excited state.

An elaborate discussion of the relaxation pathways in carotenoids can be found in.^23^ In the work of Koyama and co-workers, in the apolar solvent *n*-hexane lycopene exhibited four lifetimes of 0.20, 0.10, 0.45, and 3.9 ps^11^ and the states were assigned to 1B_u_^+^, 3A_g_^–^, 1B_u_^–^ and 2A_g_^–^ with ν_1_ bands around 1580 and 1783 cm^–1^. It should be noted that the current results differ significantly from those of Koyama and co-workers. In addition, it remains unclear how their observed spectroscopic signatures relate to the molecular nature of the proposed excited state cascade. In the polar solvent THF we observe that the ν_1_ bands of the excited states gradually shift to 1800 cm^–1^, and that singlet excited states with lifetimes of 0.07, 0.17, 0.8, and 3.3 (with a 14% subpopulation of 5) ps are present. In addition, we observe the S* state decaying with 7 ps, which in^15^ is attributed to a ground state intermediate. We consider the shapes of the TA and FSRS SADS of S1vh, S1h, and S1 (red, blue and green in Figure 2F and Figure 4C,D) similar and attribute the differences to vibrational cooling. Thus, we assign all these states to 2A_g_^–^ and find no evidence for the involvement of 3A_g_^–^ and 1B_u_^–^. It is expected that theoretical computations, analogous to those in^24^ can shed more light on the interpretation of our results.

The estimated Damped Oscillation Associated Spectrum parameters (*DOAS*_*n*_ (ω), *φ*_*n*_ (ω), *ω*_*n*_,*γ*_*n*_) are shown in Figure S15 and Figure S16. The estimated frequencies are ≈913, ≈1154 and ≈1511 cm^–1^. The latter two provide a precise estimation of the location of the ν_2_ and ν_1_ Raman resonances in the GS. The estimated damping rates are related to the ≈1 ps duration of the Raman pulse and to the width of the Lorentzian Raman lines.^10^ The DOAS description of the complex CA provides a middle ground where the data can be well described (Figure 3) with interpretable parameters, enabling the resolution of the properties of the first two excited states (black and red curves in Figure 4) with lifetimes within the fwhm of the IRF. The target model allows for a decomposition of the FSRS data (Figure S17 and Figure S18) where the contributions from the CA and the excited states at each wavenumber are resolved.

The simultaneous target analysis of the FSRS and TAS methodology developed here is expected to contribute to a more complete understanding of the ground and excited state properties of carotenoids (especially regarding the short-lived S2, the multiple S1, and the S* states) and other chromophores with strong Raman signals.

## Methods

The FSRS spectroscopy setup, employing the spectral watermark method,^5,25^ represents an upgraded version compared to that described in previous work. In this design, two independent 1 kHz chirped pulse amplifiers were employed, both seeded with femtosecond pulses from a shared Ti:sapphire oscillator. To initiate a photoreaction, a 200 nJ, ≈50 fs, 480 nm actinic pump (Ap) was generated by an optical parametric amplifier (OPA). Simultaneously, a 1450 nm signal beam from a second OPA system was focused on a moving CaF_2_ plate to generate a white light supercontinuum serving as the probe (Pr). The 770–795 nm femtosecond pulses from the second amplifier passed through a home-built pulse shaper, creating a series of frequency-locked picosecond pulses as the Raman pump (Rp), with an energy of 3 μJ. The Raman pulses were generated in the interval from 770 to 795 nm and resulting Raman spectra represent an average of the signals from all Raman experiments conducted over this interval. Simultaneous recording of the TAS, FSRS Stokes gain, and FSRS anti-Stokes loss in a smart configuration with synchronized detectors provides a comprehensive data set for each laser shot. Per 100 laser pulses, the Rp was blocked 4 times, and the Ap was blocked 50 times. This resulted in 48 transient Raman experiments and 2 transient absorption experiments. This approach is more effective, since TA signals are typically orders of magnitude stronger and require much shorter acquisition times and thus maximizes the information retrieval from a single excitation pulse, and allows for online verification of FSRS probing the same state as determined by prior TA experiments. The transient absorption signal was subtracted from the resulting transient Raman spectra and all spectrally shifted Raman signals were recombined based on the spectral calibration. In the time-resolved study, both Stokes and Anti-Stokes FSRS and TA of lycopene in THF were recorded simultaneously from 750 to 1900 cm^–1^ for 421 logarithmically spaced delays between Ap and Rp/Pr. The optical density with the FSRS spectroscopy setup was ≈1 at 480 nm. The concentration was 30 mg/L corresponding to ≈60 μM. This high OD is needed to achieve a sufficient SNR in the FSRS experiments. There were no signs of aggregation which has been double-checked by measuring the TAS on a dedicated TA setup.

This dedicated, home-built TA setup was constructed around a 1-kHz amplified Ti:sapphire laser system (Femtopower, Spectra Physics) that served as the primary source of both the pump and the probe beam. As the actual probe beam (1/e^2^-diameter in focus: 50 μm) served the white-light supercontinuum generated in an argon-filled hollow-core fiber (Ultrafast Innovations). The pump beam (480 nm, 5 nJ per pulse, 1/e^2^-diameter in focus: 95 μm) was generated in a NOPA (TOPAS, Light Conversion). The OD_480 nm_ of lycopene in THF was equal to 0.1, 0.3, and 1.0 (in a 1 mm-thick optical cell). In addition, the CA of the solvent THF was measured. The timing between the pump and probe pulses was controlled by a mechanical delay line. The relative polarization of the pump and probe beams was set to the magic angle. Both beams were chopped by optomechanical choppers allowing for shot-to-shot basis detection of the TA signal corrected for pump scattering and detector dark current. The probe transmitted through the sample was spectrally dispersed in a home-built dual-channel prism spectrometer and detected by a CCD camera (Entwicklungsbüro Stresing). The probe spectral fluctuations negatively affecting the TA signal quality were corrected using the approach described in.^26^ The sample was kept in a 1 mm-thick optical cell, the position of which was scanned in the transversal plane during the experiment to replenish the fresh sample.

The general target analysis methodology has been described in.^8^ The observed Time Resolved Spectrum (TRS) depends upon time *t* and wavenumber ω, *TRS*(*t*,ω). According to the superposition principle it can be described by a linear combination of the contributions of the different states. It is assumed that *N*_*states*_ electronically excited states are present in the system under study, with populations *c*_*l*_^*S*^ (*t*)(superscript S stands for species), and species’ spectral properties, the Species Associated Difference Spectra (*SADS*_*l*_ (ω)). Heuristically, we describe the resonance-like prezero signals with the help of *N*_*osc*_ exponentially damped oscillations, where the frequency of each oscillation depends upon the distance from the Raman resonance. Together these Raman resonances constitute the ground state Raman resonance spectrum of lycopene in the solvent THF. The amplitude of a damped oscillation cos((ω – *ω*_*n*_)*t*)exp(−*γ*_*n*_*t*) as a function of the detection wavenumber constitutes a Damped Oscillation Associated Spectrum (*DOAS*_*n*_ (ω)) with an accompanying wavenumber-dependent phase *φ*_*n*_ (ω). The part of the data not described by the parametric model is termed the *residual*(*t*,ω). Thus, we arrive at the following formula for the superposition model of the observations
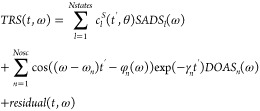


For the model-based analysis of the prezero signals we defined a new building block for damped oscillations ∑_*n* = 1_^*Nosc*^ cos((ω – *ω*_*n*_)*t*′ – *φ*_*n*_ (ω))exp(−*γ*_*n*_*t*′)*DOAS*_*n*_(ω) in the modular, extendable problem solving environment pyGlotaran.^27,28^*t*′ indicates that the actual model function still has to take into account the convolution with the IRF.^8^ The enormous complexity of this target analysis can only be mastered with the help of the structured problem solving environment pyGlotaran,^28^ which enables simultaneous target analysis of different groups of data (Stokes and Anti-Stokes FSRS and TA and visible TA, five FSRS data sets, and four dedicated TA data sets, with 3208378 data points in total), linking the kinetic and the IRF properties of the five FSRS data sets and thereby estimating 27 nonlinear parameters and 60453 conditionally linear parameters with the help of nonlinear least-squares. The relative precision of the estimated parameters is better than 10%. To reduce the number of free parameters the IRF width of the FSRS experiments is linked to the IRF width of the visible TA experiment. To avoid interference with the first SADS we omit the IRF associated difference spectrum (IRFAS) from the superposition model of the TA experiments in Figure S7(8) and aim for a good correspondence between the visible TA SADS measured from ≈687–930 nm (Figure S7I,J) and the TA SADS measured without the Raman pulse during the FSRS measurement (Figure S7G,H). In addition to the damped oscillations we employ a scatter component to describe the intense dispersive signal around the Raman line maximum visible in Figure 1B around 0 ps and 913 cm^–1^: *irf*(*t*)*IRFAS*(ω), where *irf*(*t*) represents the IRF and *IRFAS*(ω) is assumed to be zero above 1100 cm^–1^ to avoid interference with the first SADS.

## Supporting Information Available

Eighteen figures demonstrating the quality of the simultaneous fit of all data sets, details of the DOAS, and decompositions of the FSRS data (PDF)
