# Supplementary material for: Target Analysis Resolves the Ground and Excited State Properties from Femtosecond Stimulated Raman Spectra
Source: J Phys Chem Lett. 2024 Sep 6;15(37):9397–404. doi: 10.1021/acs.jpclett.4c01555 (PMC11417988; doi:10.1021/acs.jpclett.4c01555)
Supplement: Supplementary file 1 — jz4c01555_si_001.pdf [file jz4c01555_si_001.pdf]

Supporting Information

# Target Analysis Resolves The Ground And Excited State Properties From Femtosecond Stimulated Raman Spectra

*Ivo H.M. van Stokkum*[1], *Joris J. Snellenburg*[1], *Petra Chrupková*[2], *Jakub Dostal*[2], *Sebastian Weigand*[1], *Jörn Weißenborn*[1], *John T.M. Kennis*[1], *Miroslav Kloz*[2]

[1]Department of Physics and Astronomy and LaserLaB, Faculty of Science, Vrije Universiteit Amsterdam, De Boelelaan 1081, 1081 HV, Amsterdam, The Netherlands

[2]The Extreme Light Infrastructure ERIC, ELI Beamlines Facility, Za Radnicí 835, 252 41 Dolní Břežany, Czech Republic

## Structures

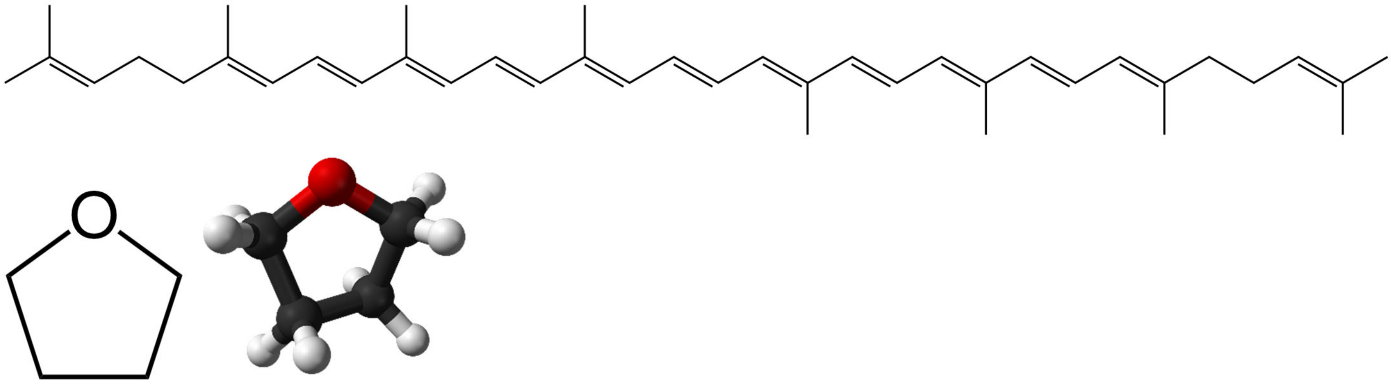

Figure S 1. (top) Backbone structure of lycopene $C_{40}H_{56}$, a bright red carotenoid hydrocarbon found in tomatoes and other red fruits and vegetables. (bottom) Structure of the polar solvent tetrahydrofuran $(CH_2)_4O$ (THF)

# Fit quality of TAS measured on a dedicated TA setup

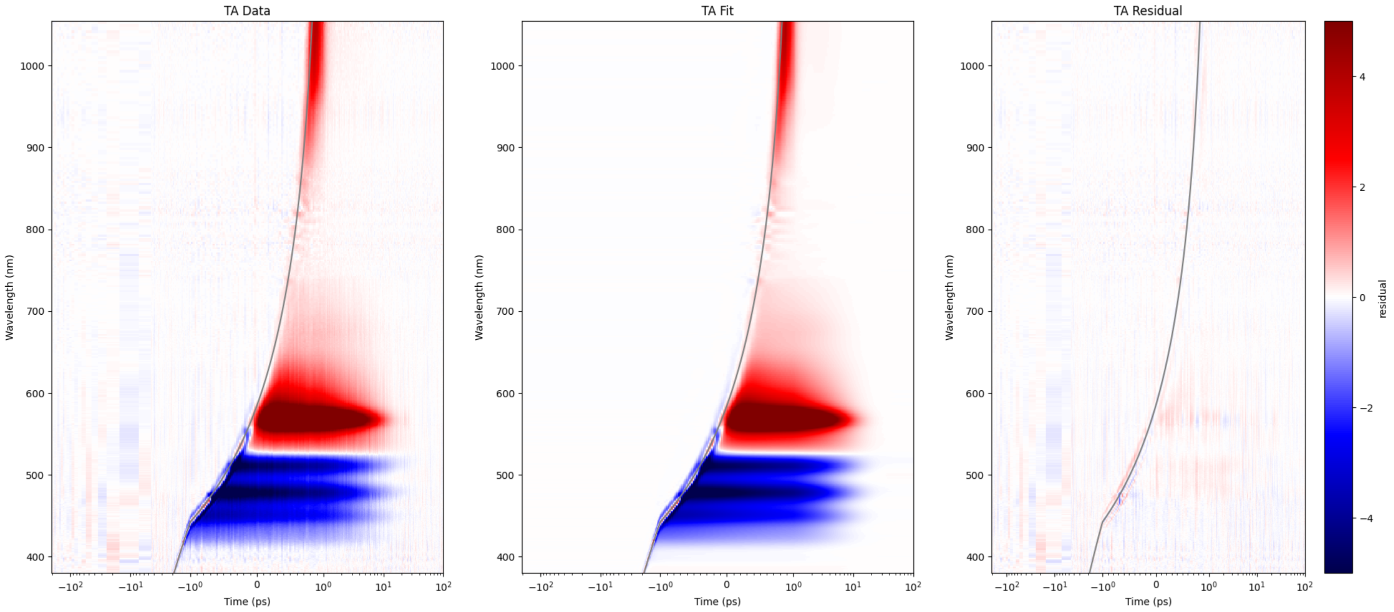


Figure S 2. Target analysis of TA of lycopene in THF with $OD_{480nm}$ equal to 0.1 (in mOD) measured on a dedicated TA setup, note the qualitative and quantitative agreement. From left to right: data, fit and residual. In grey the estimated dispersion curves (the location of the maximum of the IRF). Note that the time axis is linear from -1 to 1 ps and logarithmic elsewhere.

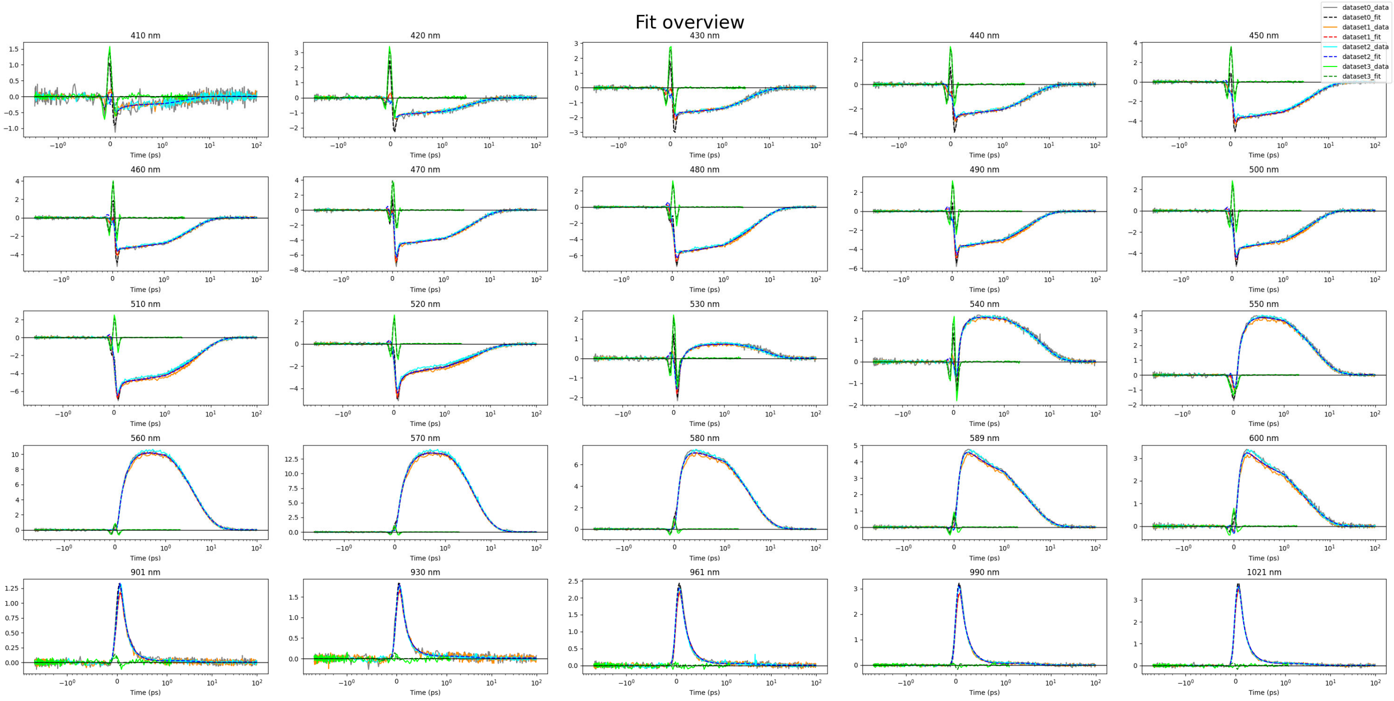


Figure S 3. Fit quality at 25 selected wavelengths (indicated in the title of each panel) of TA measured on a dedicated TA setup. Three different samples of lycopene in THF have been measured with $OD_{480nm}$ equal to 0.1, 0.3 and 1.0 (dataset 0, 1, and 2, respectively). Key: scaled data (grey, orange and cyan, in mOD), fit line in dashed black, red and blue. The green line represents the CA in THF measured with a pump power of 5 nJ per pulse, which was used in all experiments, and the fit of the CA is in dashed dark green.

# Overview of visible TA

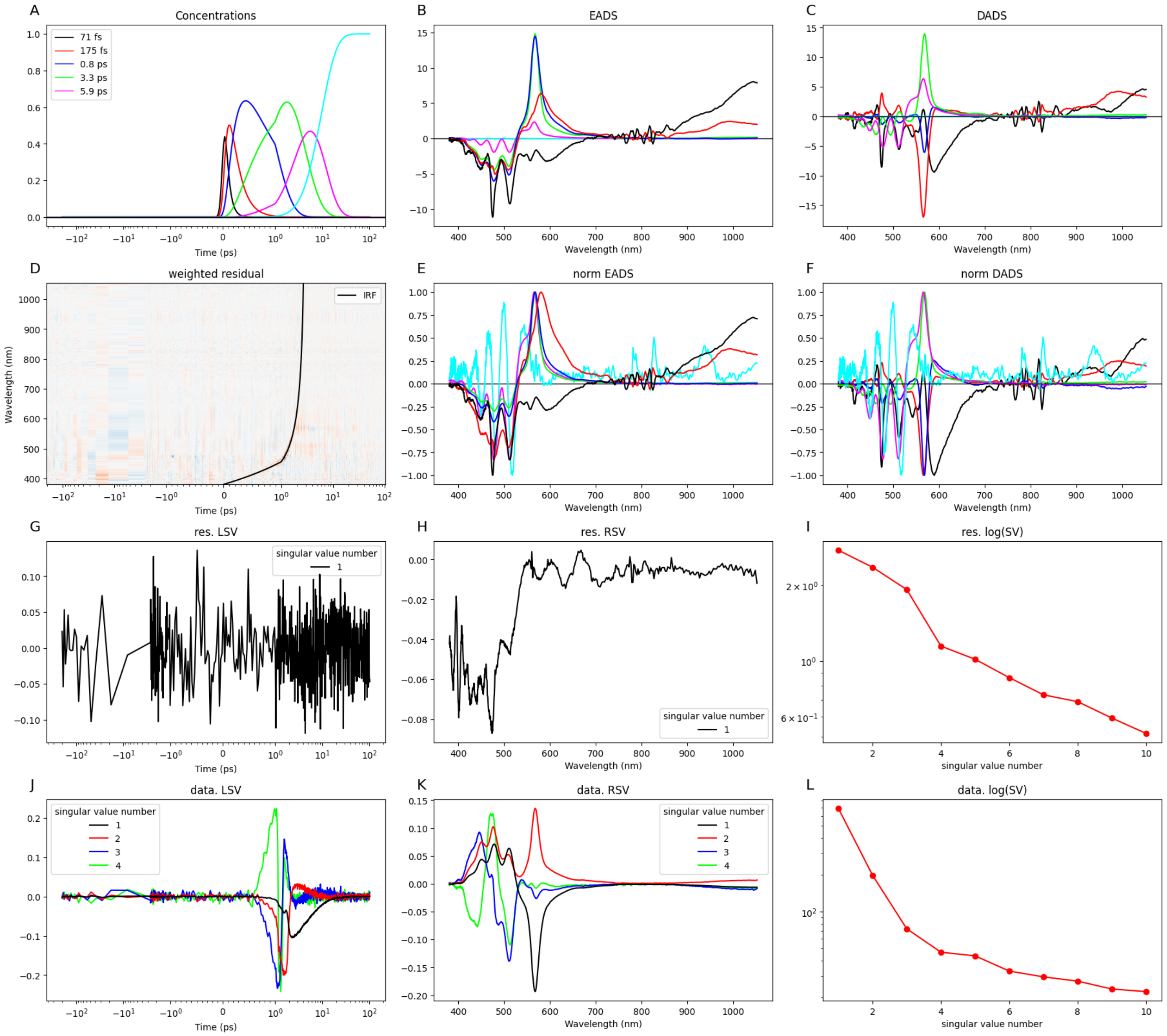


Figure S 4. Overview of visible TA of lycopene in THF with $OD_{480nm}$ equal to 0.1: Concentrations of the global analysis (A), EADS (B) and normalized EADS (E). Note that IRFAS have been used. Lifetimes : 71, 175 fs, 0.8, 3.3 and 5.9 ps, long lived. (C,F) are the DADS and normalized DADS. No trends in the residuals are visible in (D). The black dispersion curve in D indicates the wavelength dependence of the maximum of the IRF. (G-I) The singular value decomposition of the matrix of residuals: (G) first left singular vector, showing no trends (H) first right singular vector, showing dominant amplitude around 480 nm (I) screeplot of the singular values. (J-L) The singular value decomposition of the matrix of the data: (J) first four left singular vectors, (K) first four right singular vectors, (L) screeplot of the singular values.

# Overview of visible TA measured on the FSRS setup

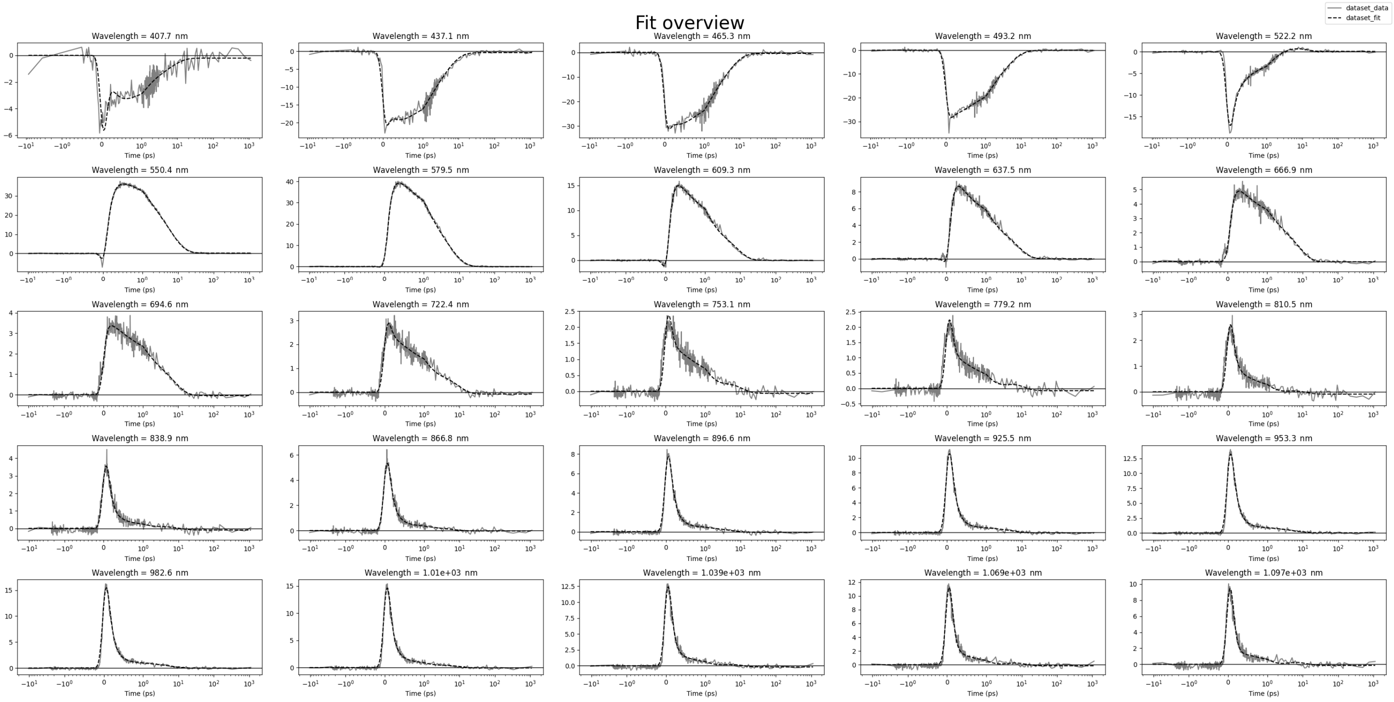


Figure S 5. Fit quality at 25 selected wavelengths of TA measured on the FSRS setup with $OD_{480nm}$ equal to 1 (grey, in mOD), fit line in dashed black.

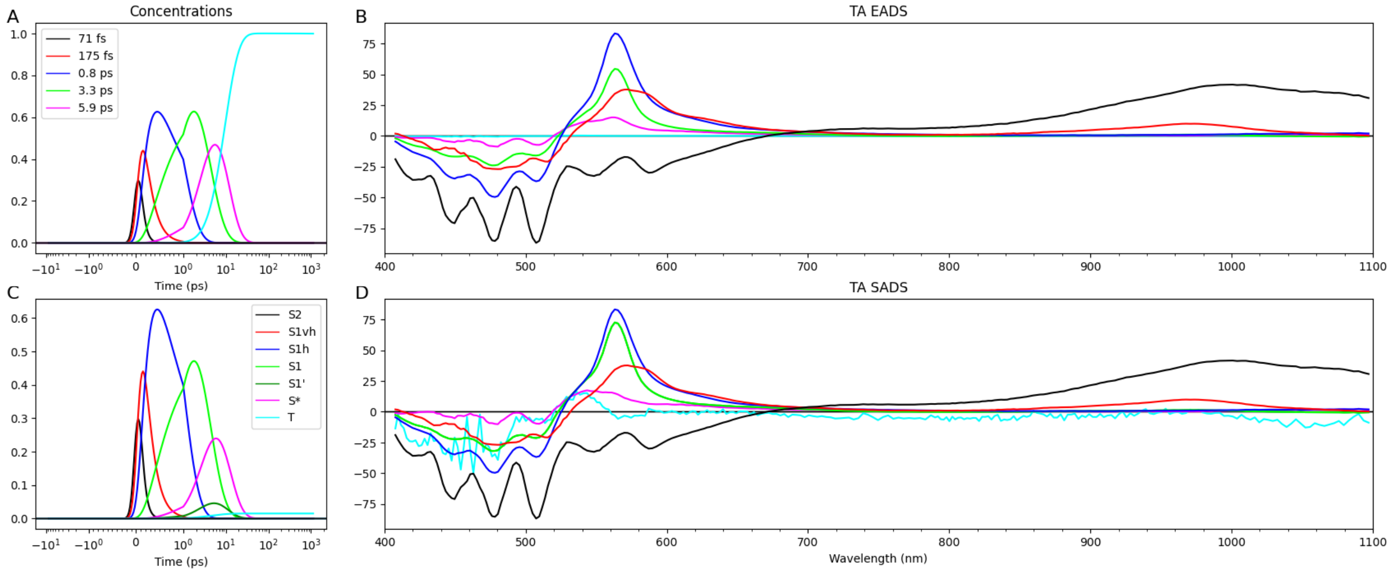


Figure S 6. Global and target analysis of TAS of lycopene in THF with $OD_{480nm}$ equal to 1. Concentrations (A,C) of a sequential scheme without losses (Figure 2A) and of a target kinetic scheme (Figure 2D). Legends in (A,C) indicate lifetimes and species, l.l. is long-lived; S1vh is very hot S1, S1h is hot S1, S1’ is a more slowly decaying subpopulation of S1 with identical SADS, T is triplet. Estimated EADS (B) and SADS (D, both in mOD) using the kinetic schemes from Figure 2A and D.

# Overview of concentrations and all EADS

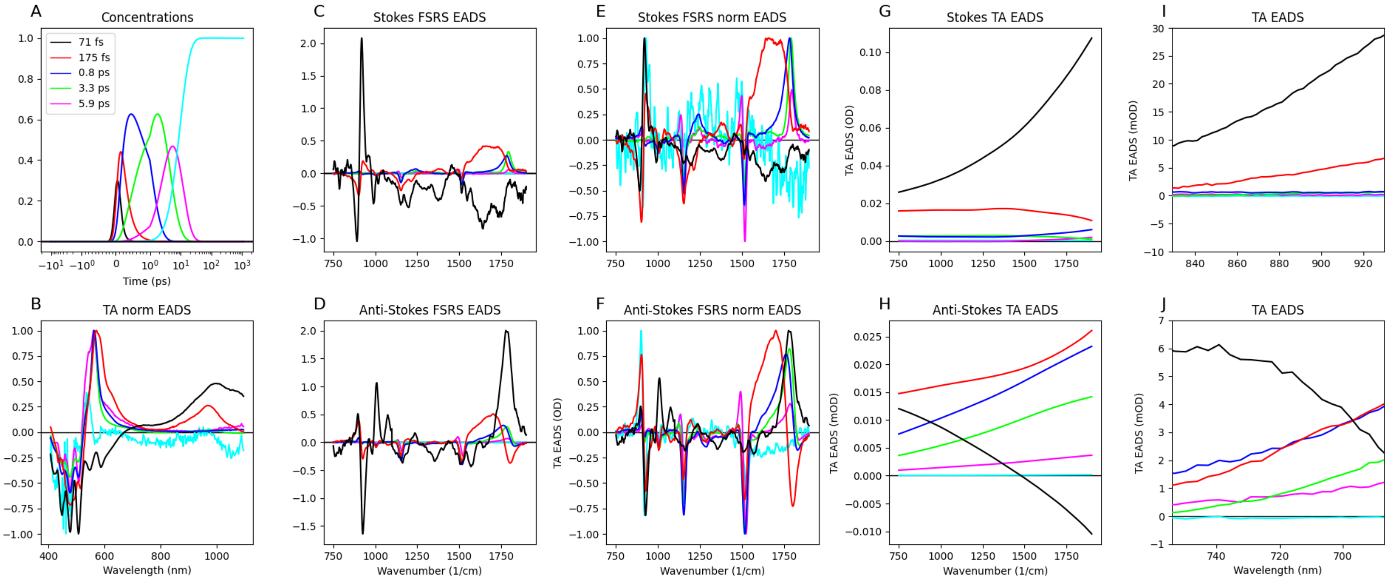


Figure S 7. Concentrations (A) and EADS (B-J). G,H are TA EADS with the same dispersion as the FSRS. I,J are selections of the TA EADS of panel B. Legend for all panels in (A). Lifetimes: 71, 175 fs, 0.8, 3.3 and 5.9 ps, long lived (cyan). For a detailed overview of the FSRS global analysis and the (norm) EADS, see Figure S 13. and Figure S 14. , and Figure S 4. for the visible TA global analysis. The quality of the fit is demonstrated in Figure S 12. (FSRS), Figure S 9. (TA in FSRS regions), and Figure S 3. (visible TA). With Raman excitation wavelength of ≈790 nm the Stokes range 600-1900 $cm^{-1}$ corresponds to ≈829-930 nm (I), and the Anti-Stokes range 600-1900 $cm^{-1}$ corresponds to ≈687-754 nm (J).

# Fit quality of TAS in the Stokes and the Anti-Stokes regions

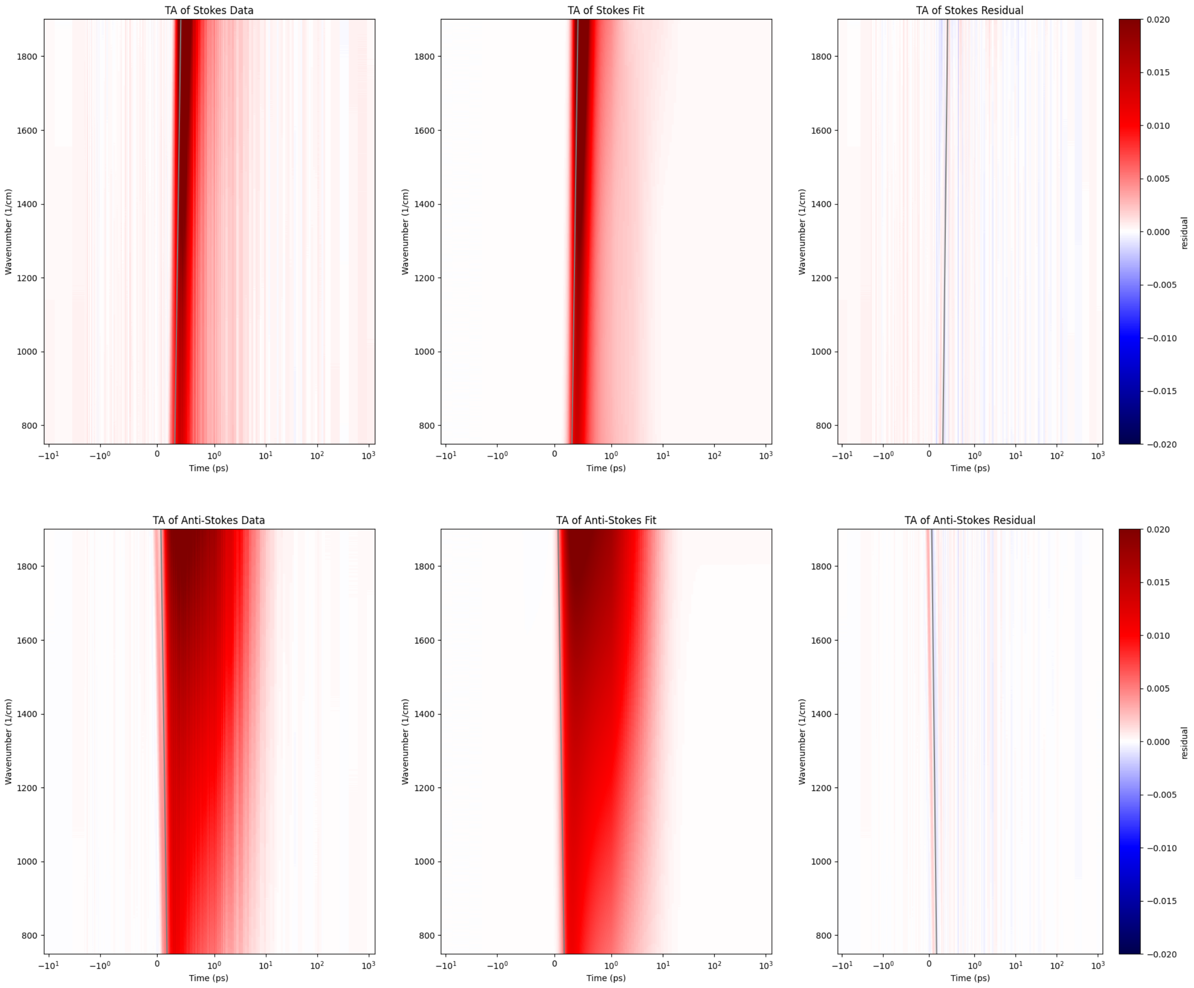


Figure S 8. Target analysis of TA in the FSRS region of lycopene in THF (in OD), note the qualitative and quantitative agreement. From left to right: data, fit and residual. In grey the estimated dispersion curves (the location of the maximum of the IRF).

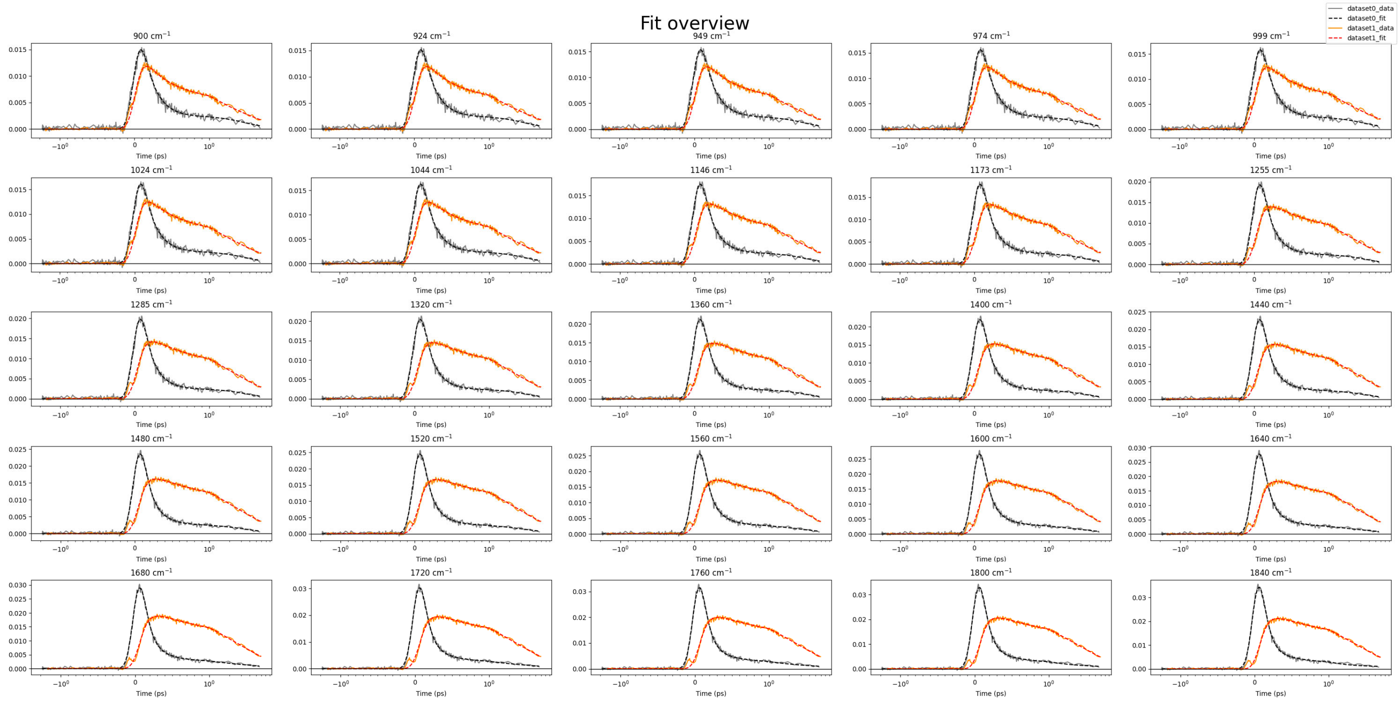


Figure S 9. Fit quality at 25 selected wavenumbers of TA of Stokes (grey) and Anti-Stokes (orange) in the FSRS regions, fit lines in dashed black and dashes red respectively.

# Overview of Stokes TA of FSRS

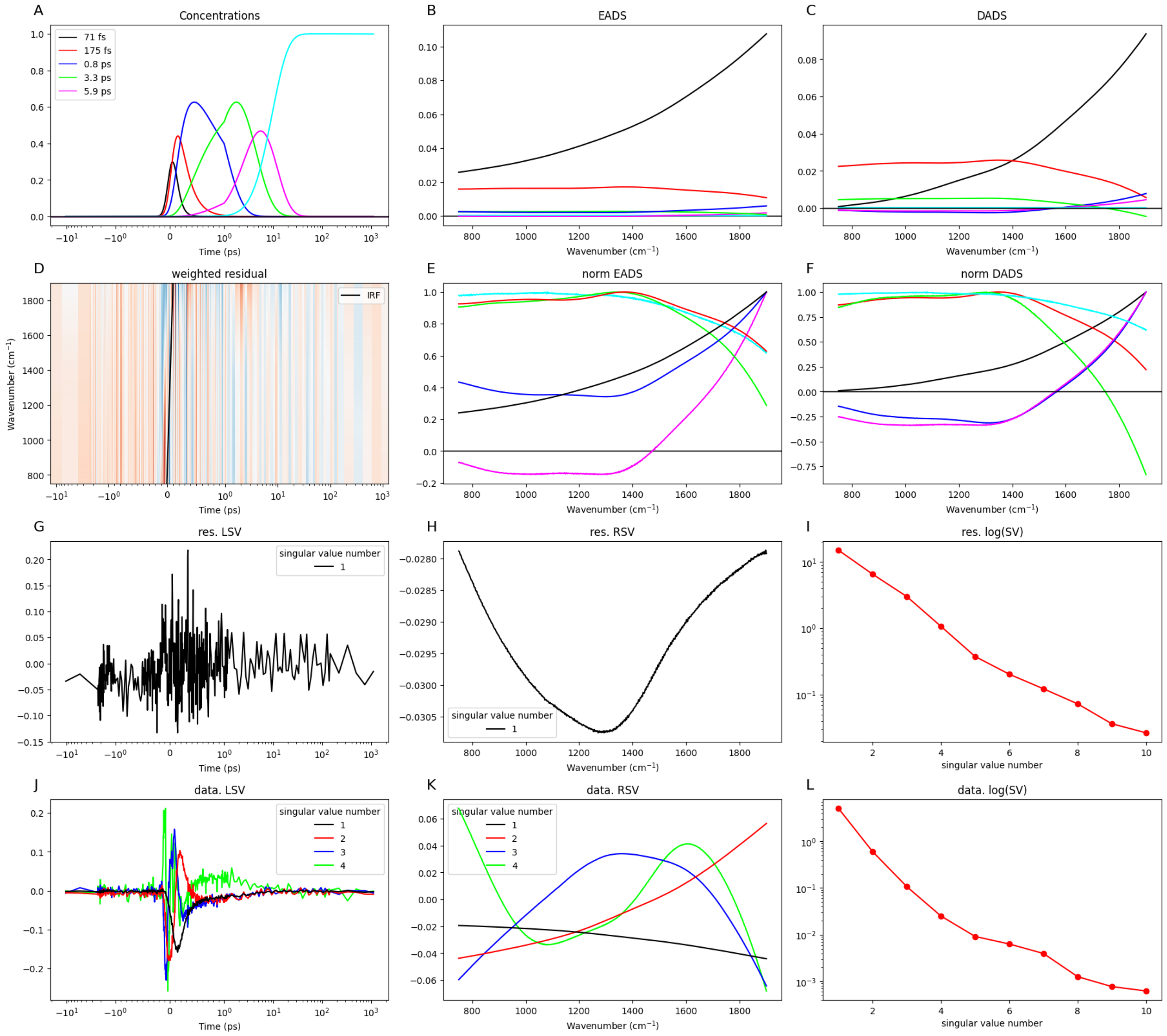


Figure S 10. Overview of Stokes TA of FSRS of lycopene in THF: Concentrations of the global analysis (A), EADS (B) and normalized EADS (E). Note that IRFAS have NOT been used. Legend for all panels in (A). Lifetimes: 71, 175 fs, 0.8, 3.3 and 5.9 ps, long lived (cyan). (C,F) are the DADS and normalized DADS. No trends in the residuals are visible in (D). The black dispersion curve in D indicates the wavenumber dependence of the maximum of the IRF. (G-I) The singular value decomposition of the matrix of residuals: (G) first left singular vector, showing no trends (H) first right singular vector, showing dominant amplitude around 1300/cm (I) screeplot of the singular values. (J-L) The singular value decomposition of the matrix of the data: (J) first four left singular vectors, (K) first four right singular vectors, (L) screeplot of the singular values.

# Overview of Anti-Stokes TA of FSRS

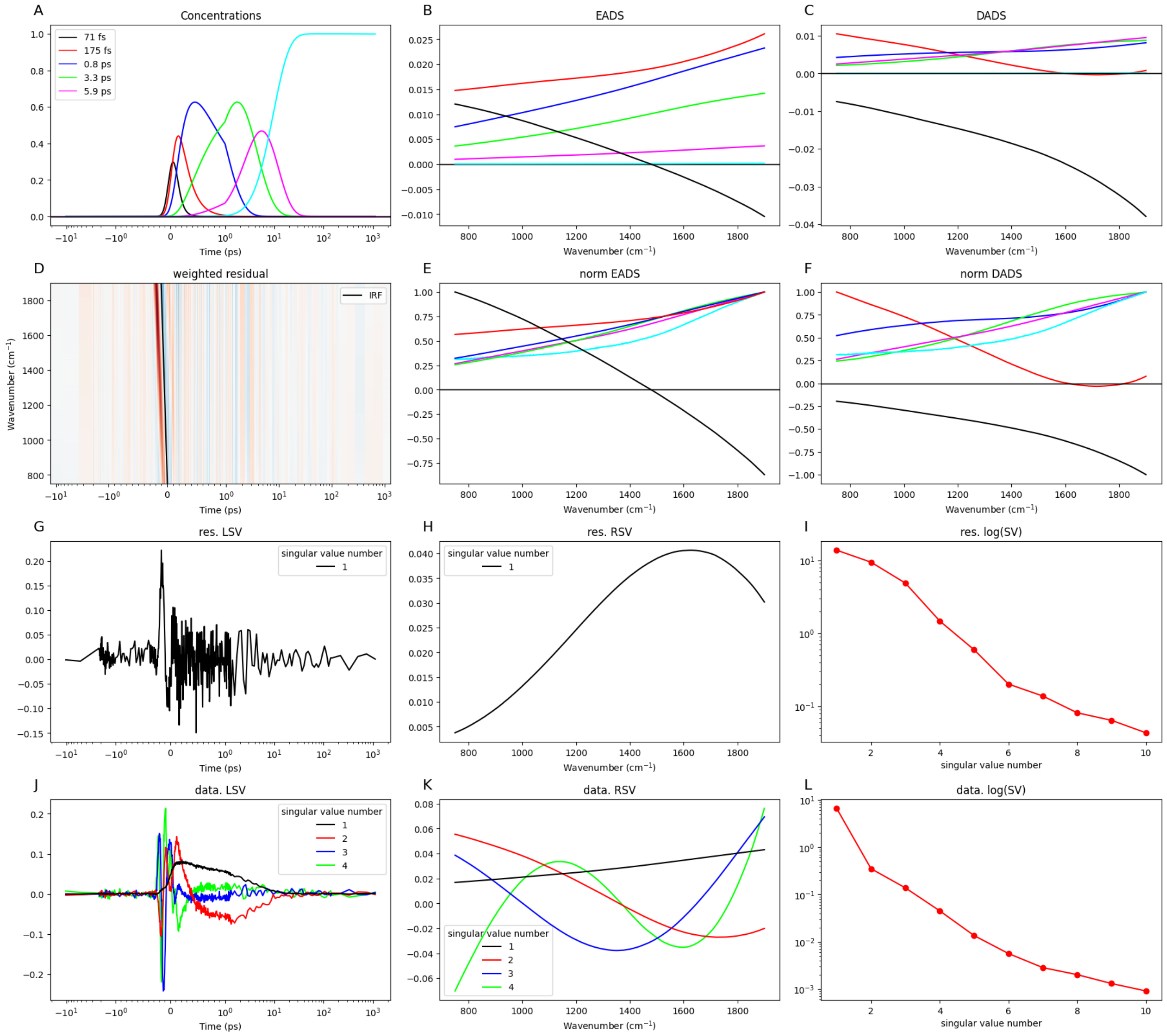


Figure S 11. Overview of Anti-Stokes TA of FSRS of lycopene in THF: Concentrations of the global analysis (A), EADS (B) and normalized EADS (E). Note that IRFAS have NOT been used. Legend for all panels in (A). Lifetimes: 71, 175 fs, 0.8, 3.3 and 5.9 ps, long lived (cyan). (C,F) are the DADS and normalized DADS. Small trends in the residuals before time zero are visible in (D). The black dispersion curve in D indicates the wavenumber dependence of the maximum of the IRF. (G-I) The singular value decomposition of the matrix of residuals: (G) first left singular vector, showing small trends in the residuals before time zero (H) first right singular vector, showing dominant amplitude around 1600/cm (I) screeplot of the singular values. (J-L) The singular value decomposition of the matrix of the data: (J) first four left singular vectors, (K) first four right singular vectors, (L) screeplot of the singular values.

# Fit quality of FSRS

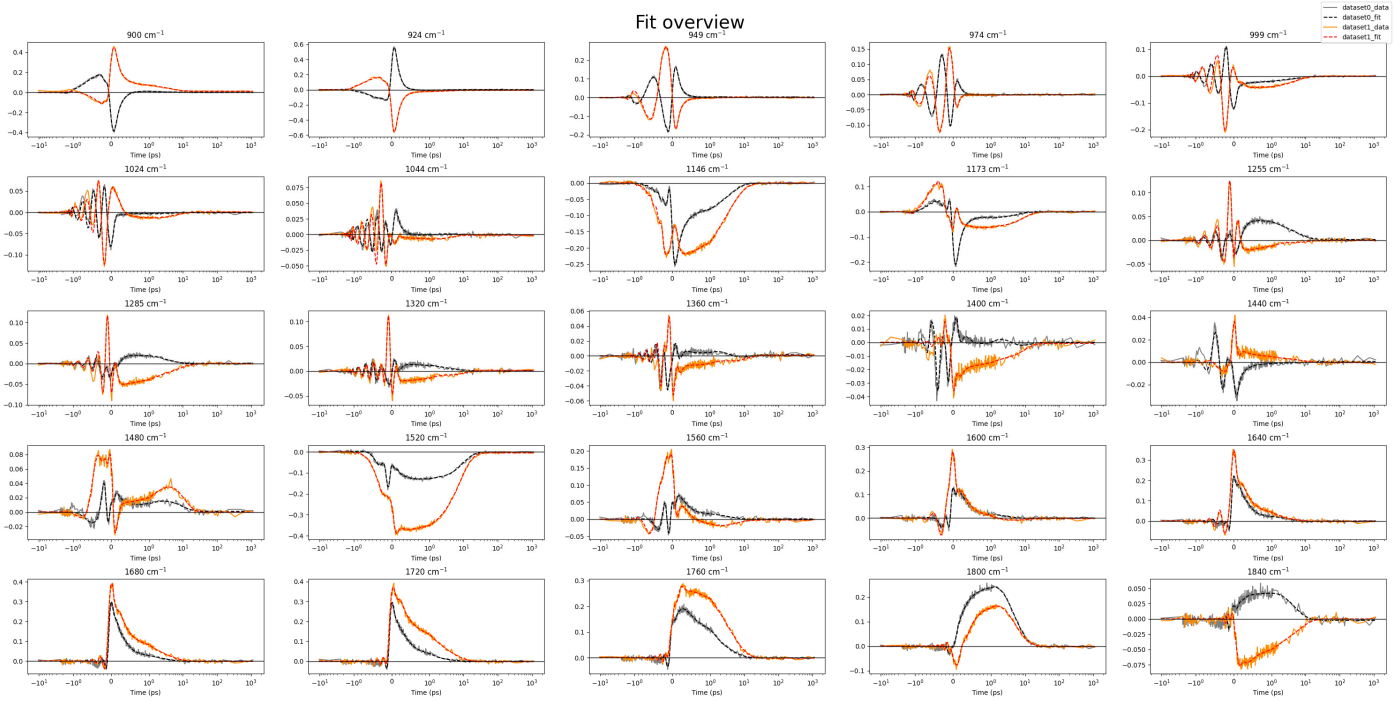


Figure S 12. Fit quality at 25 selected wavenumbers of Stokes (grey) and Anti-Stokes (orange) FSRS, fit lines in dashed black and dashes red respectively.

# Overview of Stokes FSRS

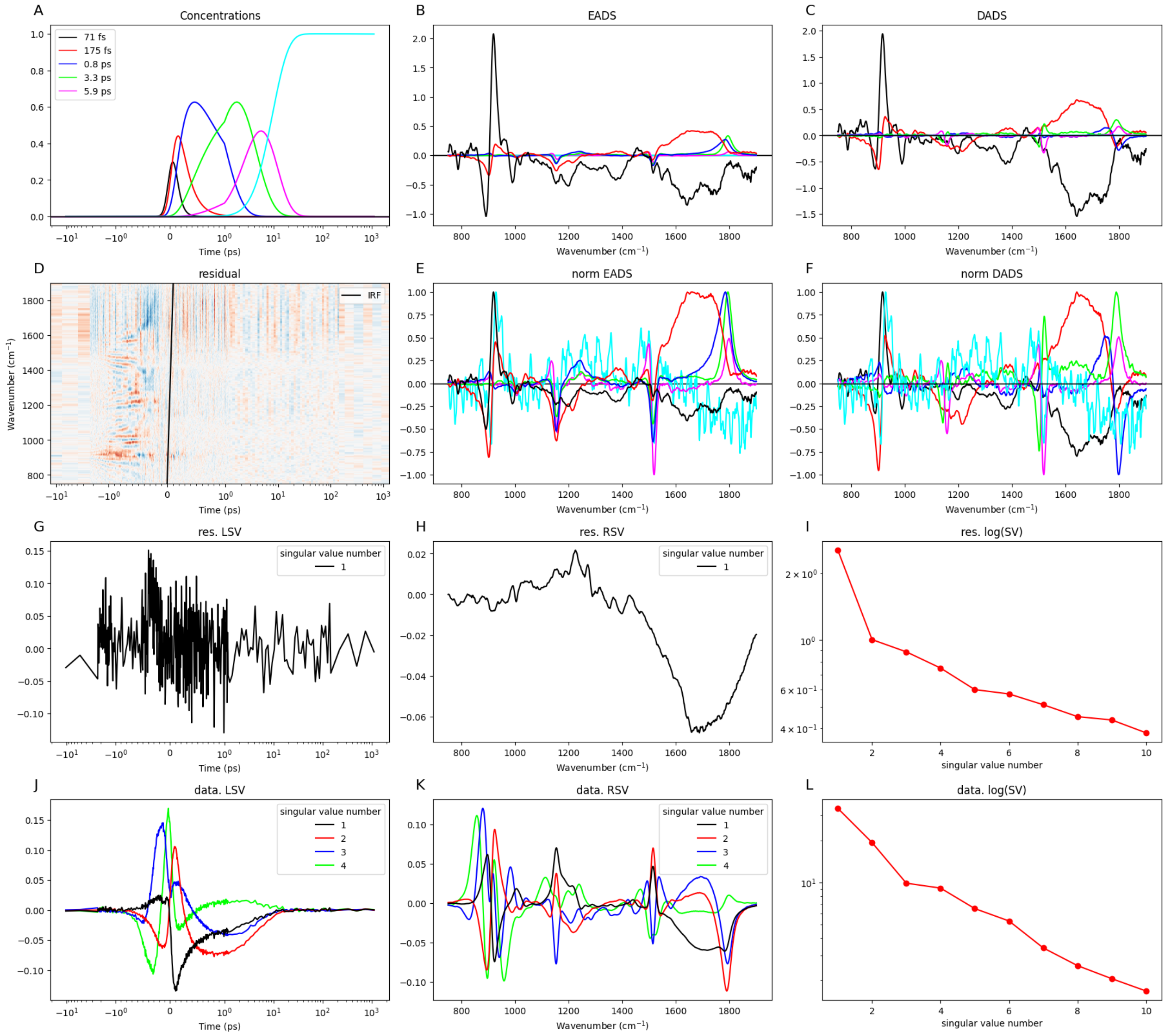


Figure S 13. Overview of Stokes FSRS of lycopene in THF: Concentrations of the global analysis (A), EADS (B) and normalized EADS (E). Note that IRFAS have NOT been used. Legend for all panels in (A). Lifetimes: 71, 175 fs, 0.8, 3.3 and 5.9 ps, long lived (cyan). (C,F) are the DADS and normalized DADS. Small trends in the residuals before time zero are visible in (D). The black dispersion curve in D indicates the wavenumber dependence of the maximum of the IRF. (G-I) The singular value decomposition of the matrix of residuals: (G) first left singular vector, showing only small trends (H) first right singular vector, showing dominant amplitude around 1700/cm (I) screeplot of the singular values. (J-L) The singular value decomposition of the matrix of the data: (J) first four left singular vectors, (K) first four right singular vectors, (L) screeplot of the singular values.

# Overview of Anti-Stokes FSRS

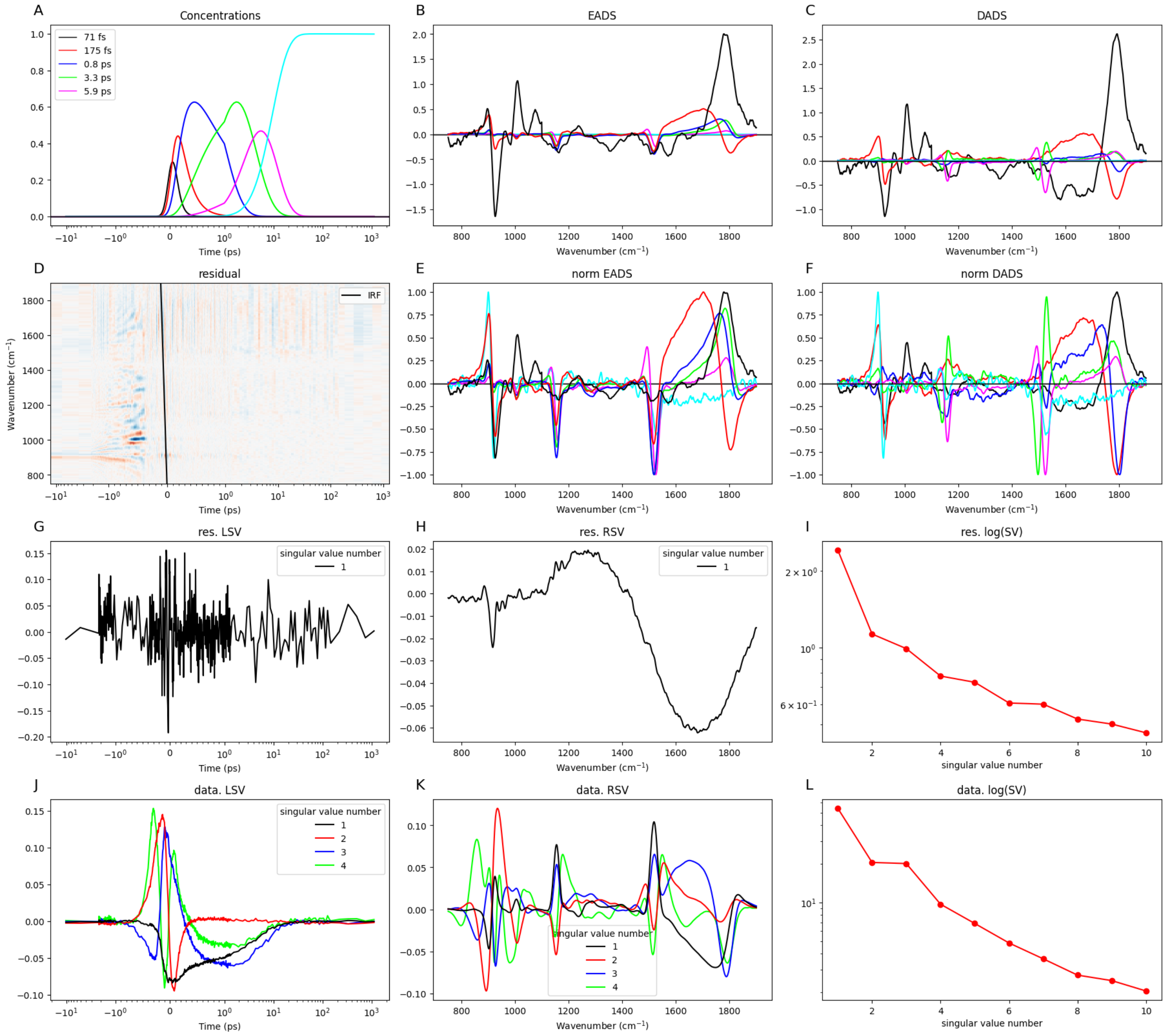


Figure S 14. Overview of Anti-Stokes FSRS of lycopene in THF: Concentrations of the global analysis (A), EADS (B) and normalized EADS (E). Note that IRFAS have NOT been used. Legend for all panels in (A). Lifetimes: 71, 175 fs, 0.8, 3.3 and 5.9 ps, long lived (cyan). (C,F) are the DADS and normalized DADS. Small trends in the residuals before time zero are visible in (D). The black dispersion curve in D indicates the wavenumber dependence of the maximum of the IRF. (G-I) The singular value decomposition of the matrix of residuals: (G) first left singular vector, showing no trends (H) first right singular vector, showing dominant amplitude around 1700/cm (I) screeplot of the singular values. (J-L) The singular value decomposition of the matrix of the data: (J) first four left singular vectors, (K) first four right singular vectors, (L) screeplot of the singular values.

# DOAS and Phases

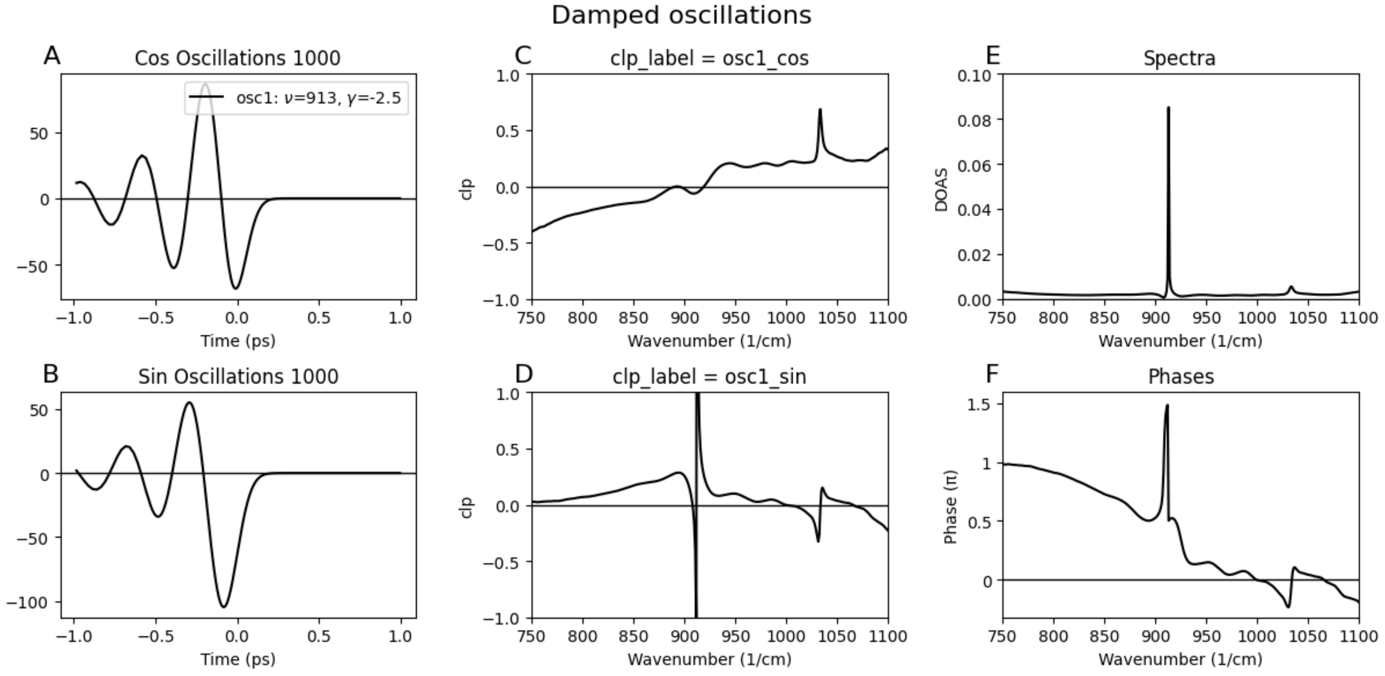


Figure S 15. Overview of the THF damped oscillations with resonance frequency 913 $cm^{-1}$ (A) damped cosine far away from resonance at 1000 $cm^{-1}$. The frequency is 1000-913=87 $cm^{-1}$ damped with 2.5 $ps^{-1}$. (B) Sine of 87 $cm^{-1}$ damped with 2.5 $ps^{-1}$. (C,D) estimated cosine and sine amplitudes (clp = conditionally linear parameter) (E,F) DOAS and phase.

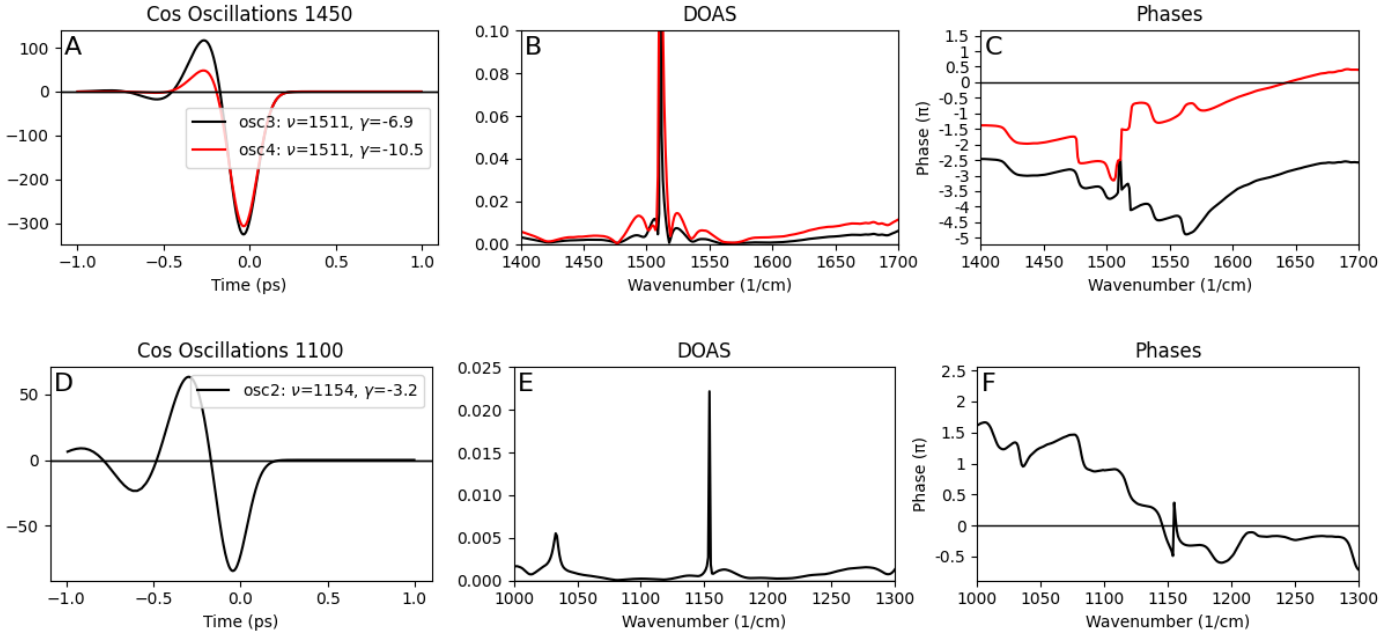


Figure S 16. Overview of the damped oscillations attributed to the ν1 (A-C) and ν2 (D-F) bands of lycopene (A,D) damped cosines far away from resonance at frequencies indicated in the panel labels. (B,E) DOAS (C,F) Phase. No zero constraints are imposed on the DOAS.

# Decomposition of the Stokes data

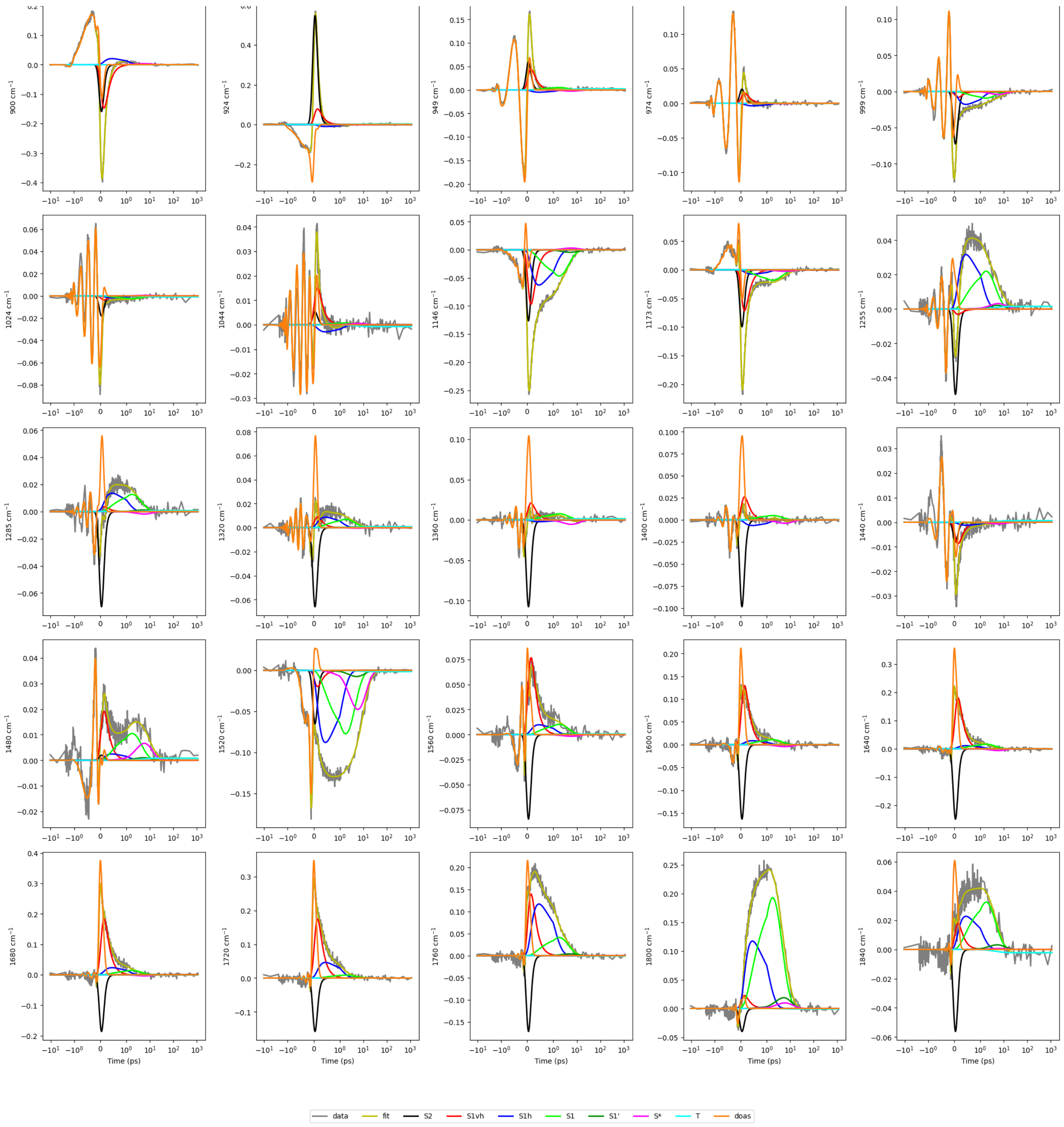


Figure S 17. Decomposition of the Stokes data (grey, mOD) and fit (yellow) at 25 selected wavenumbers between 900 and 1840 $cm^{-1}$, indicated in the ordinate labels. Key: black S2, red very hot S1, blue hot S1, green S1, dark green S1' (a more slowly decaying subpopulation of S1 with identical SADS), magenta S*, cyan T. The CA/PFID signal (labelled doas, orange) overlaps around time zero with S2 (black).

# Decomposition of the Stokes data around the THF resonance frequency

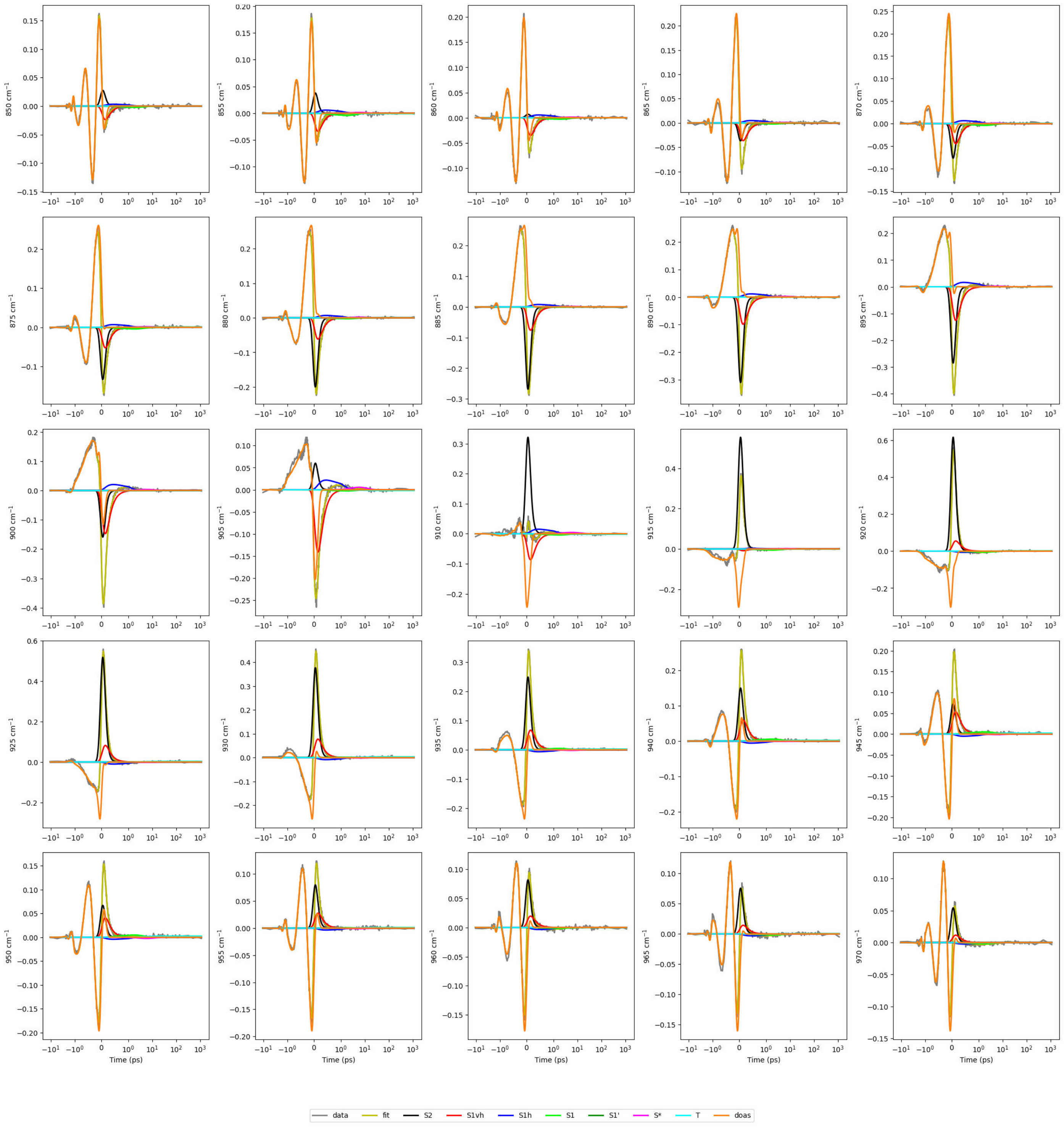


Figure S 18. Decomposition of the Stokes data (grey, mOD) and fit (yellow) at 25 selected wavenumbers between 850 and 970 $cm^{-1}$, i.e. around the THF resonance frequency of 913 $cm^{-1}$. Key: black S2, red very hot S1, blue hot S1, green S1, dark green S1' (a more slowly decaying subpopulation of S1 with identical SADS), magenta S*, cyan T. The CA/PFID signal (labelled doas, orange) overlaps around time zero with S2 (black).
